# Self-Renewal of Single Mouse Hematopoietic Stem Cells Is Reduced by JAK2V617F Without Compromising Progenitor Cell Expansion

**DOI:** 10.1371/journal.pbio.1001576

**Published:** 2013-06-04

**Authors:** David G. Kent, Juan Li, Hinal Tanna, Juergen Fink, Kristina Kirschner, Dean C. Pask, Yvonne Silber, Tina L. Hamilton, Rachel Sneade, Benjamin D. Simons, Anthony R. Green

**Affiliations:** 1 Cambridge Institute for Medical Research, University of Cambridge, Cambridge, United Kingdom; 2 Department of Haematology, University of Cambridge, Cambridge, United Kingdom; 3 Stem Cell Institute, University of Cambridge, Cambridge, United Kingdom; 4 Cavendish Laboratory, Department of Physics, University of Cambridge, Cambridge, United Kingdom; 5 Department of Haematology, Addenbrooke's Hospital, Cambridge, United Kingdom; Baylor College of Medicine, United States of America

## Abstract

Recent descriptions of significant heterogeneity in normal stem cells and cancers have altered our understanding of tumorigenesis, emphasizing the need to understand how single stem cells are subverted to cause tumors. Human myeloproliferative neoplasms (MPNs) are thought to reflect transformation of a hematopoietic stem cell (HSC) and the majority harbor an acquired V617F mutation in the JAK2 tyrosine kinase, making them a paradigm for studying the early stages of tumor establishment and progression. The consequences of activating tyrosine kinase mutations for stem and progenitor cell behavior are unclear. In this article, we identify a distinct cellular mechanism operative in stem cells. By using conditional knock-in mice, we show that the HSC defect resulting from expression of heterozygous human JAK2V617F is both quantitative (reduced HSC numbers) and qualitative (lineage biases and reduced self-renewal per HSC). The defect is intrinsic to individual HSCs and their progeny are skewed toward proliferation and differentiation as evidenced by single cell and transplantation assays. Aged JAK2V617F show a more pronounced defect as assessed by transplantation, but mice that transform reacquire competitive self-renewal ability. Quantitative analysis of HSC-derived clones was used to model the fate choices of normal and JAK2-mutant HSCs and indicates that JAK2V617F reduces self-renewal of individual HSCs but leaves progenitor expansion intact. This conclusion is supported by paired daughter cell analyses, which indicate that JAK2-mutant HSCs more often give rise to two differentiated daughter cells. Together these data suggest that acquisition of JAK2V617F alone is insufficient for clonal expansion and disease progression and causes eventual HSC exhaustion. Moreover, our results show that clonal expansion of progenitor cells provides a window in which collaborating mutations can accumulate to drive disease progression. Characterizing the mechanism(s) of JAK2V617F subclinical clonal expansions and the transition to overt MPNs will illuminate the earliest stages of tumor establishment and subclone competition, fundamentally shifting the way we treat and manage cancers.

## Abstract 2

In this study, single cell assays and mathematical modeling demonstrate that a single oncogenic point mutation can negatively affect hematopoietic stem cells while leaving progenitor cell expansion intact.

## Author Summary

Recent descriptions of the existence of significant heterogeneity in normal stem cells and cancers have altered our understanding of tumorigenesis, emphasizing the need to understand how single stem cells are subverted to cause tumours. In this study, we focus on understanding the stem cell defect that results from a mutation in the JAK2 tyrosine kinase gene, which is present in the majority of patients with myeloproliferative neoplasms (MPNs), a group of clonal bone marrow diseases that are characterised by the overproduction of mature blood cells and increased frequency of leukaemia development. By using single-cell assays and mathematical modeling, followed by the individual assessment of daughter cells from single HSCs, we identify a distinct cellular mechanism that differentially affects stem cell and progenitor cell expansion. Specifically, we show that this single point mutation can negatively affect HSCs while leaving progenitor cell expansion intact. Characterising the mechanisms that link JAK2 mutations with clonal expansions that eventually lead to development of MPNs will inform our understanding of the earliest stages of tumour establishment and of the competition between subclones of proliferating progenitor/stem cells. These findings have direct relevance to all cancers of a suspected stem cell origin.

## Introduction

The hematopoietic system produces multiple types of specialized blood cells and its lifelong maintenance relies upon hematopoietic stem cells (HSCs) [1]. One of their most intriguing characteristics is the execution of balanced fate choices in order to maintain themselves and to provide the correct numbers and types of progeny to ensure homeostasis [2]. When this balance is perturbed, malignancy can result from the clonal dominance of HSCs that have acquired differentiation and/or proliferation abnormalities [3]. In order to understand the balance between self-renewal and differentiation throughout the lifetime of an organism, mathematical modeling has given seminal insight in epithelial systems where lineage tracing and defined organ structure have permitted such analyses [4],[5].

Driver mutations within the HSC compartment are associated with several hematological malignancies. The myeloproliferative neoplasms (MPNs) are of particular interest for several reasons. Chronic phase MPNs are frequently diagnosed at an early presymptomatic stage of disease and are associated with overproduction of morphologically normal mature cells [6],[7]. There is no differentiation block and no need for the neoplastic clone to overcome tissue barriers, hypoxia, or other environmental hurdles. As a consequence, MPNs provide a window onto some of the earliest stages of malignancy that are inaccessible in other cancers. Moreover, they are experimentally tractable since they readily permit clonal analysis and are chronic diseases, thereby facilitating the dissection of clonal evolution [8]–[10].

In 2005, a single acquired mutation, JAK2V617F, was reported to be present in most MPN patients [11]–[14]. Subsequently, several mouse models have provided important insights into the biological consequences of mutant JAK2 [15]. More recently, several groups have developed JAK2V617F knock-in models to study the effect of physiological levels of JAK2V617F [16]–[19]. Our model conditionally expresses a single copy of human JAK2V617F under the control of the mouse Jak2 regulatory elements following pIpC injection prior to 6 wk of age [19]. These mice (hereafter called JAK2^V617F^) develop a phenotype strongly resembling human JAK2V617F-positive ET with modest increases in platelet and hemoglobin levels together with transformation to more severe disease (splenomegaly with erythrocytosis or myelofibrosis) in ∼10% of mice. In both competitive and noncompetitive transplantation experiments, whole bone marrow (BM) from JAK2^V617F^ mice showed a disadvantage compared to wild-type (WT) littermate controls [19] reminiscent of oncogene-induced senescence in other cancers.

Here we have studied the balance of fate choice in highly purified JAK2^V617F^ HSCs in limiting dose transplantations and single-cell analyses combined with a mathematical modeling approach and direct assessment of first division progeny. We demonstrate that HSCs are reduced in number, exhibit reduced self-renewal on a per cell basis, and generate progeny that display increased proliferation and differentiation. Moreover, quantitative analyses of single HSCs indicate that JAK2V617F reduces HSC self-renewal whilst leaving intact expansion of early progenitors. Together our results indicate that JAK2V617F alone compromises HSC self-renewal and is insufficient to sustain long-term clonal expansion in the absence of additional mutations.

## Results

### JAK2V617F Reduces HSC Self-Renewal and Produces Variable Lineage Outputs

**Figure 1:**
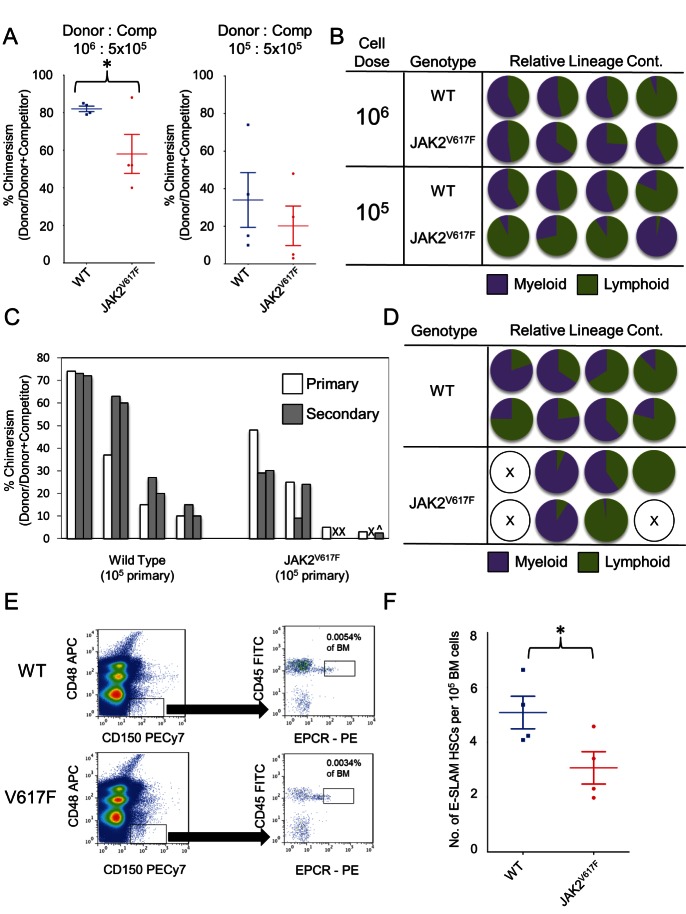
JAK2V617F induces a loss of self-renewal activity and HSC numbers and leads to a lineage bias when limited HSCs are transplanted. (A) Relative chimerism following transplantation of 10^6^ or 10^5^ JAK2^V617F^ or wild type (WT) whole bone marrow (BM) 6–10 months post-pIpC along with 5×10^5^ whole BM competitor cells into eight recipient mice. Average chimerism was lower in mice receiving JAK2^V617F^ cells (*p* = 0.03). (B) The relative myeloid (purple) versus lymphoid (green) contribution in each of the recipient animals were determined by calculating a ratio between the contribution to the myeloid compartment [Donor GM/(Donor GM+Competitor GM)] and lymphoid compartment [Donor BT/(Donor BT+Competitor BT)]. (C) Relative chimerism in primary peripheral blood (PB) (white bars) in the eight animals receiving 10^5^ cells compared to levels of PB chimerism in the 16 secondary recipients (two per primary animal, grey bars) in two independent transplantation experiments. An “X” represents a recipient that showed less than 1% chimerism at 24 wk posttransplantation, and a ∧ represents a recipient that only had contribution to the lymphoid lineages. (D) The relative myeloid (purple) versus lymphoid (green) contribution in each of the secondary recipient animals were determined by calculating a ratio between the contribution to the myeloid and lymphoid compartments as in Figure 1B. (E) The FACS isolation strategy for CD45^+^/EPCR^+^/CD48^−^/CD150^+^ (E-SLAM) cells. The panels are gated on viable white blood cells and show E-SLAM gates for WT (top) and JAK2^V617F^ (bottom). (F) The frequency of E-SLAM HSCs per 10^5^ viable bone marrow (BM) cells in four WT and four JAK2^V617F^ mice 6–10 mo following pIpC injection from four independent experiments. The frequency is reduced in JAK2^V617F^ animals (*p* = 0.0288).

Competitive transplantation studies were performed to investigate the HSC defect observed in JAK2^V617F^ mice. In order to study the effect of transplanting limiting numbers of HSCs and investigate potential lineage biases, transplantations were performed using low numbers of donor cells. When transplanted with 10^5^ donor BM cells, mice receiving either WT or JAK2^V617F^ cells displayed reduced PB chimerism compared to those receiving the corresponding 10^6^ cell dose (Figure 1A). Importantly, whereas mice receiving 10^6^ JAK2^V617F^ cells retained a balanced production of myeloid and lymphoid lineages, those receiving 10^5^ JAK2^V617F^ cells exhibited marked lineage skewing (Figure 1B) as determined by the relative production of myeloid and lymphoid elements by donor and competitor cells.

When transplanted into secondary animals (Figure 1C), BM cells from primary recipients of 10^5^ WT cells successfully repopulated secondary recipient mice. BM cells from primary recipients of 10^5^ JAK2^V617F^ cells also repopulated secondary recipients, but compared to competitor cells, JAK2^V617F^ cells produced fewer progeny, with several secondary recipients displaying no detectable contribution or only low levels of lymphoid cells. Secondary recipients of JAK2^V617F^ BM cells again showed lineage skewing, with four of five positive recipients showing >90% bias toward either the myeloid or lymphoid lineage (Figure 1D) compared to zero of eight recipients of WT primary BM. As there are reduced HSC numbers in the JAK2^V617F^ mice, the observed lineage biases (Figure 1B and 1D) may reflect transplantation of only a few HSCs, thereby revealing lineage-biased HSCs that have been previously described [20],[21]. Taken together, these results demonstrate that HSCs from JAK2^V617F^ BM display reduced self-renewal and that lineage biases emerge when limiting numbers of HSCs are present.

### JAK2^V617F^ E-SLAM HSCs Are Less Numerous and Have a Reduced Self-Renewal Capacity per HSC

We previously reported reduced numbers of c-Kit^+^, Sca1^+^, lineage negative (KSL) cells in JAK2^V617F^ BM 6 mo following pIpC injection [19]. While the KSL population contains most HSCs, only one in 30 can repopulate an irradiated mouse at 2 mo posttransplantation [22],[23], and so we focused these studies on highly purified CD45^+^EPCR^+^CD48^−^CD150^+^ (E-SLAM) HSCs, 56% of which produce multilineage clones at 4 mo posttransplantation [24]. In JAK2^V617F^ mice, E-SLAM HSC numbers were reduced 2-fold (*p* = 0.029) compared to WT littermate controls (Figure 1E and F).

To ascertain whether E-SLAM HSCs from JAK2^V617F^ mice were less functional than normal controls, 10 donor E-SLAM HSCs were transplanted alongside competitor BM cells and secondary transplantations were performed using BM from all the primary recipients showing even trace amounts of donor-derived repopulation. For mice receiving WT E-SLAM HSCs, four of five primary transplant recipients and four of four secondary transplant recipients displayed long-term contributions by WT test cells at 16–24 wk (Table 1). In contrast, mice receiving JAK2^V617F^ E-SLAM HSCs gave rise to significantly less long-term repopulation (*p* = 0.019), with just one of five primary recipients showing a long-term contribution and three other recipients showing <1% of JAK2^V617F^ donor cells at 16–24 wk posttransplantation (Table 1). Moreover, BM from the primary recipients of JAK2^V617F^ donor HSCs did not give rise to any long-term contribution in secondary recipients (Table 1). JAK2^V617F^ E-SLAM HSCs showed no difference in their ability to home to the bone marrow within the first 36 h posttransplantation compared to E-SLAM HSCs from WT littermate controls (Figure S1D and Methods S1). Collectively, these data demonstrate that E-SLAM HSC numbers are reduced in JAK2^V617F^ animals and are functionally compromised in long-term serial transplantation assays.

**Table 1:** Primary and secondary transplantation of 10 highly purified JAK2^V617F^ or wild type E-SLAM HSCs.

Dose	Wild type		JAK2^V617F^	
	Mouse	% Chimerism	Mouse	% Chimerism
Primary (10 HSCs)	A	15	F	2
	B	10	G	0*
	C	47	H	0*
	D	38	I	0*
	E	0	J	0
Secondary (6×10^6^)	From A	3, 7	From F	0, 0
	From B	3, 2	From G	0, 0
	From C	80, 28	From H	0, 0
	From D	30, 36	From I	0, 0

Donor bone marrow was harvested and E-SLAM HSCs isolated as shown in Figure 1E. Ten E-SLAM HSCs from JAK2^V617F^ mice 6–10 mo following pIpC injection or their wild-type littermate controls were transplanted into each of five irradiated recipients alongside 200,000 competitor cells. The numbers of successful transplantations and their respective chimerism levels are shown above for both primary (10 E-SLAM HSCs) and secondary (6×10^6^ bone marrow cells from the primary animal) transplantations. A successful transplantation was defined as those showing >1% donor peripheral blood chimerism (out of donor+competitor) at 16–24 wk and at least 0.5% peripheral blood chimerism within each lineage (GM, B, and T) at some point over the 16-wk period. For each primary mouse, two secondary transplantations were performed and the original parent is indicated. Mice showing trace amounts of repopulation (<1%) are marked with an asterisk (*).

### JAK2^V617F^ E-SLAM HSCs Have a Survival Advantage In Vitro and Generate Larger, More Differentiated Clones

**Figure 2:**
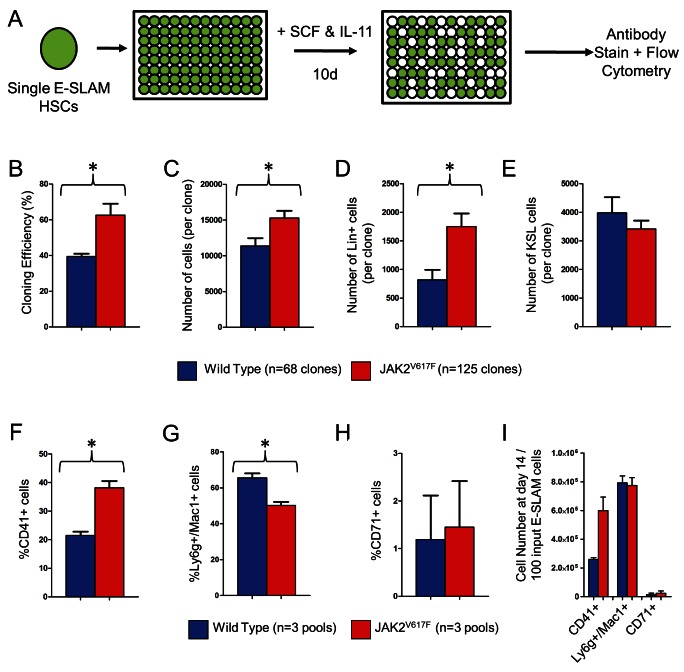
JAK2^V617F^ HSCs have an initial survival advantage and make larger, more differentiated clones. (A) Schematic for single cell in vitro cultures. Individual CD45^+^/EPCR^+^/CD48^−^/CD150^+^ (E-SLAM) cells, obtained from mice 6–10 mo following pIpC injection, were sorted into single wells and cultured for 10 d in 300 ng/mL SCF and 20 ng/mL IL-11 in four independent experiments. (B) The average cloning efficiency was higher (*p* = 0.05) for JAK2^V617F^ (red bars) versus wild type (blue bars) cells and was measured by counting the number of sorted events that give rise to a colony after 10 d. (C) The average number of cells per clone was higher (*p* = 0.016) in JAK2^V617F^ cells. JAK2^V617F^ HSCs give rise to more differentiated cells (*p* = 0.006) as measured by the expression of one or more of a panel of lineage markers (CD5, Mac1, CD19, B220, Ly6g, 7-4, or Ter119, panel D) and expression of c-Kit and Sca1 as a surrogate for stem/progenitor cell number (E). Fourteen-day cultures of 100–400 E-SLAM HSCs in SCF+IL-11 followed by flow cytometric analysis of the cells show that, by proportion, JAK2^V617F^ HSCs make more CD41^+^ (*p* = 0.003, F), and less Ly6g/Mac1^+^ cells (*p* = 0.008, G) than wild-type controls in three independent experiments. The proportion of CD71^+^ cells generated was not changed (H). (I) The absolute numbers of Ly6g/Mac1^+^ and CD71^+^ cells generated were not different, but the number of CD41^+^ cells produced was increased approximately 2-fold (*p* = 0.023).

To study the stem cell defect in individual HSCs, we used a single-cell in vitro culture system previously reported to maintain numbers of long-term repopulating cells [20],[25]. Single E-SLAM HSCs (*n* = 720), obtained from JAK2^V617F^ mice or WT littermate controls, were assessed for survival, early kinetics of cell division, proliferation, and differentiation state (Figure 2). Compared to WT E-SLAM HSCs, the number of wells giving rise to a 10-d clone from JAK2^V617F^ E-SLAM HSCs was increased by approximately 50% (*p* = 0.05, Figure 2B) and the average clone size was also increased (*p* = 0.016, Figure 2C). Clones derived from JAK2^V617F^ HSCs contained more differentiated cells (Lin^+^) (*p* = 0.006, Figure 2D), but did not show a significant increase in KSL cells (Figure 2E). Compared to WT equivalents, JAK2^V617F^ E-SLAM HSCs displayed similar cell cycle kinetics during their first two rounds of cell division (Figure S1A) and gave rise to similar levels of apoptotic cells after 10 d of culture (Figure S1B). These results demonstrate that JAK2^V617F^ E-SLAM HSCs are more clonogenic and give rise to more progeny expressing differentiation markers under conditions that normally maintain HSC numbers.

To investigate the differentiation potential of JAK2^V617F^ HSCs, individual pools of 100–400 E-SLAM HSCs were cultured in SCF and IL-11 and assessed at 14 d for expression of several differentiation markers. Compared to WT equivalents, JAK2^V617F^ E-SLAM HSC-derived clones contained a higher percentage of CD41^+^ cells (*p* = 0.003), a lower percentage of Ly6g^+^ and/or Mac1^+^ cells (*p* = 0.008), and similar percentages of CD71^+^ cells (Figure 2F–H). When absolute numbers of cell types were taken into account, the increase in CD41^+^ cells was even more pronounced (Figure 2I). The increased proportion and absolute number of CD41^+^ cells are consistent with a bias toward megakaryocytic differentiation, which was also observed in vivo [19]. However, the SCF and IL-11 culture conditions are specifically selected to maintain stem and progenitor cells and do not optimally support the production of more mature blood cells. Therefore, in order to further test the differentiation potential of JAK2^V617F^ E-SLAM HSCs, we undertook a series of functional assays.

### JAK2^V617F^ E-SLAM HSCs Generate More Short-Term Progenitors But Fewer Cells with Durable Self-Renewal Activity

**Figure 3:**
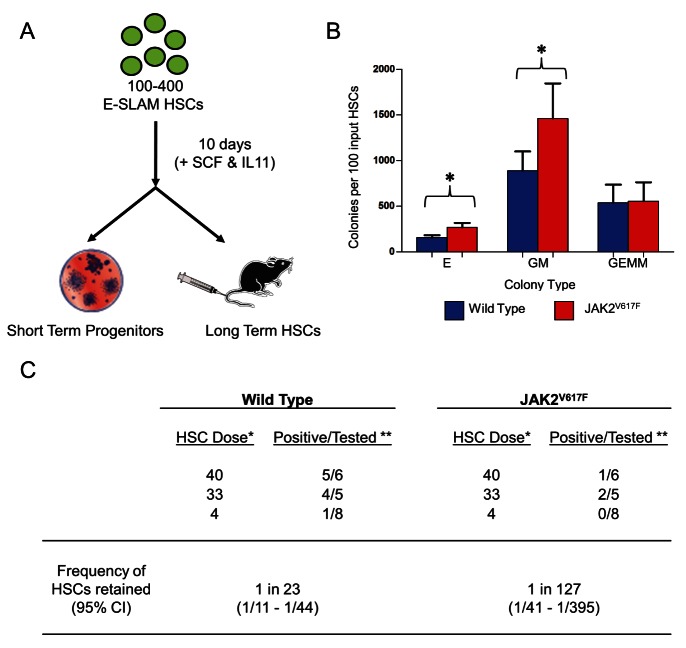
Cultured JAK2^V617F^ E-SLAM HSCs produce more short-term progenitors, but lack long-term reconstitution ability. (A) Cells derived from cultures of 100–400 E-SLAM HSCs were harvested after 10 d of culture in SCF and IL-11 and then placed in a colony-forming cell (CFC) assay to determine the number and type of progenitor cells made or were transplanted into irradiated recipients to determine whether or not long-term reconstituting ability was retained. (B) Following 10–14 d of culture in the CFC assay, colonies were scored and enumerated. Colonies were scored as either Erythroid (E), Granulocyte/Macrophage (GM), or Granulocyte/Macrophage/Erythroid/Megakaryocyte (GEMM) progenitors and are represented by bar graphs showing the mean +/– SEM of four to six biological replicates from four independent experiments. A greater number of GM (*p* = 0.009) and E (*p* = 0.007) were observed in CFCs derived from JAK2^V617F^ cultures. (C) Varying doses (40, 33, 4) of HSC starting equivalents (the proportion of the total culture that would have been made by that input number of HSCs) were transplanted to determine the frequency of cells that had retained long-term reconstituting ability in two independent experiments. This is followed by a limiting dilution analysis that estimates the frequency of HSCs retained in the culture. Cultures of JAK2^V617F^ HSCs make 5–6-fold fewer HSCs in culture compared to WT littermate controls (*p* = 0.00469). * HSC dose is defined as the number of starting equivalents that were transplanted. In the case of “40,” this is representative of transplanting all of the cells that would be generated from a 10-d culture of 40 HSCs. ** A mouse was considered to be positive if it had >1% donor chimerism at 16–24 weeks and represented at least 0.5% of each lineage (GM, B, and T) at some point over the 16-wk period.

The results described above show that JAK2^V617F^ E-SLAM HSCs make larger, more differentiated clones compared to WT E-SLAM HSCs, but did not allow us to assess the production of functional progenitors. We therefore performed short-term progenitor assays and long-term transplantation assays on the progeny of cultured E-SLAM HSCs (Figure 3A). Individual pools each containing 100–400 E-SLAM HSCs were cultured for 10 d, and the progeny were assessed by CFC assays or transplanted at different doses into irradiated recipients. In CFC assays, JAK2^V617F^ cells gave rise to significantly more BFU-E (*p* = 0.007) and CFU-GM (*p* = 0.009), but displayed no increase in the number of CFU-GEMM (Figure 3B). The CFC culture conditions do not detect lymphoid progenitors, and so these results do not exclude expansion of lymphoid-committed progenitors.

In transplantation experiments, the 10-d progeny of 40, 33, or 4 HSC starting equivalents were injected into a total of 38 irradiated recipient mice to assess self-renewal during the in vitro culture. While cultures derived from WT E-SLAM HSCs repopulated most recipients using 40 (5/6) or 33 (4/5) E-SLAM starting equivalents, those derived from JAK2^V617F^ E-SLAM HSCs contained substantially fewer cells capable of long-term repopulation (1/6 and 2/5 recipients repopulated, respectively). Overall, JAK2^V617F^ E-SLAM HSCs retained 5–6-fold fewer HSCs over 10 d compared to WT equivalents (*p* = 0.005, Figure 3C). These data demonstrate that JAK2^V617F^ E-SLAM HSCs underwent markedly fewer HSC self-renewal divisions, but produced similar numbers of primitive progenitors (CFU-GEMM) and generated significantly more erythroid and granulocyte/macrophage (GM) progenitors.

### JAK2^V617F^ Mice Progress to More Severe Disease, But in Those That Do Not, E-SLAM HSCs Are Not Expanded, Are Less Proliferative, and Lack Increased Clonogenicity In Vitro

To understand the normal aging of HSCs in the absence of high replicative stress (e.g., transplantation in vivo or high doses of hematopoietic cytokines in vitro), we generated a cohort of aged WT and JAK2^V617F^ mice. Six of 80 JAK2^V617F^ mice (but none of 92 littermate controls) were sacrificed, at a median of 9 mo post-pIpC injection, as a consequence of transformation to diseases resembling PV or MF. Together with mice from a smaller cohort on a mixed 129Sv/C57Bl6/J background [19], 10 mice developed either a PV phenotype or an MF phenotype. Transformation to PV was accompanied by marked erythrocytosis, splenomegaly, and low platelets, whereas MF transformation was accompanied by BM fibrosis and splenomegaly together with anemia and variable white cell and platelet counts (Table S1).

**Figure 4:**
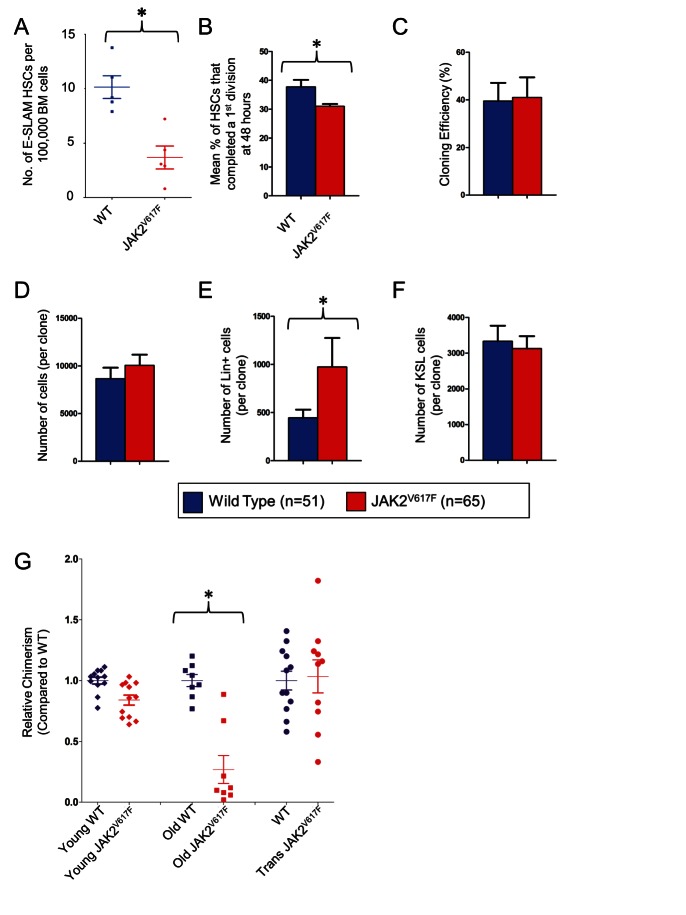
E-SLAM HSCs do not expand in old JAK2^V617F^ knock-in mice and show reduced functional ability as well as a delayed entry into the cell cycle. (A) E-SLAM HSCs were increased in frequency in wild type (∼2-fold, see Figure 1E) but not JAK2^V617F^ marrow in 18–24-mo-old mice (*n* = 10) compared to 6–10-mo-old mice resulting in a 3-fold overall reduction in E-SLAM HSCs compared to wild-type (*p* = 0.002) in three independent experiments. (B) Individual HSCs were cultured and cell counts were recorded on day 1 and day 2 to determine whether or not they had undergone a division in three independent experiments. At day 2, significantly fewer (*p* = 0.039) old JAK2^V617F^ HSCs had divided. The cloning efficiency (C), number of cells per clone (D), and number of KSL cells per clone (F) were not different, but the JAK2^V617F^ cells still produced more differentiated cell types after 10 d of culture (*p* = 0.039, E). (G) Competitive transplantation of whole bone marrow from old JAK2^V617F^ mice, transformed JAK2^V617F^ mice, and their respective WT littermate controls. Relative chimerism is calculated by measuring donor chimerism as a percentage of donor+competitor chimerism and normalized to the average of the WT contribution (set to 1). The old JAK2^V617F^ BM displays reduced chimerism (*p*<0.01), whereas transformed JAK2^V617F^ mice that have undergone transformation reacquire their self-renewal capacity.

Normally HSCs undergo several qualitative and quantitative changes with increasing age including a variably expanded phenotypically defined HSC pool, delayed proliferative responses in vitro, and reduced functional capacity in vivo as measured by transplantation of purified HSCs [26]–[28]. We therefore analyzed BM from JAK2^V617F^ mice and WT littermate controls that were 18–24 mo after pIpC injection (hereafter called old mice). In WT mice, the E-SLAM HSC compartment was ∼2-fold larger in old mice compared to younger mice (compare Figure 4A to Figure 1F). However, the same comparison in JAK2^V617F^ mice shows that the E-SLAM HSC compartment was not expanded in old mice. As a consequence, there was a 3-fold reduction (*p* = 0.002) in the frequency of E-SLAM HSCs in old JAK2^V617F^ mice compared to their WT littermate controls.

To investigate whether or not the remaining JAK2^V617F^ HSCs aged in the same way as WT HSCs, we cultured 317 single E-SLAM HSCs. Both WT and JAK2^V617F^ E-SLAM HSCs from old mice displayed decreased cell cycle entry at 48 h compared to their younger counterparts (compare Figure 4B to Figure S1C). This effect was more marked in old JAK2^V617F^ E-SLAM HSCs, which exhibited significantly delayed entry into the first cell cycle relative to old WT E-SLAM HSCs (*p* = 0.039, Figure 4B). In contrast to E-SLAM HSCs derived from younger mice, old JAK2^V617F^ E-SLAM HSCs did not show an increase in cloning efficiency (compare Figure 4C with Figure 2B) or produce significantly more cells per clone compared to WT equivalents (compare Figure 4D with Figure 2C). However, like their younger counterparts, old JAK2^V617F^ E-SLAM HSCs generated more differentiated cells (Figure 4E) and similar numbers of stem/progenitor cells per clone (Figure 4F) compared to WT littermate controls. Moreover, freshly isolated E-SLAM HSCs from old JAK2^V617F^ mice accumulated more DNA damage as determined by a greater number of γ-H2AX foci compared to WT equivalents (Figure S1G).

We next undertook competitive transplantation experiments to assess the relative repopulating ability of old JAK2^V617F^ BM and found that it was significantly reduced (*p*<0.01) at 4 mo posttransplantation (Figure 4G). Importantly, when we performed competitive transplantation experiments on the BM from JAK2^V617F^ mice that had transformed, this reduction in competitive repopulating ability was not present (Figure 4G), indicating that HSC activity is recovered in transformed mice. Moreover, in one animal, the numbers of E-SLAM HSCs was increased relative to an age-matched WT control (Figure S1E), consistent with the concept that transformation to PV is accompanied by recovery of HSC numbers.

Together, our data therefore demonstrate that in younger mice, JAK2V617F reduces HSC numbers and mutant HSCs produce more progeny than WT equivalents. By contrast, in old mice JAK2V617F is not associated with the usual expansion of the HSC compartment, and unlike their younger counterparts, mutant HSCs are no longer more productive than WT equivalents, however they recover their HSC activity in animals that transform to more severe disease.

### Quantitative Analysis of Single HSC-Derived Clones Shows That JAK2V617F Does Not Compromise Progenitor Cell Self-Renewal

**Figure 5:**
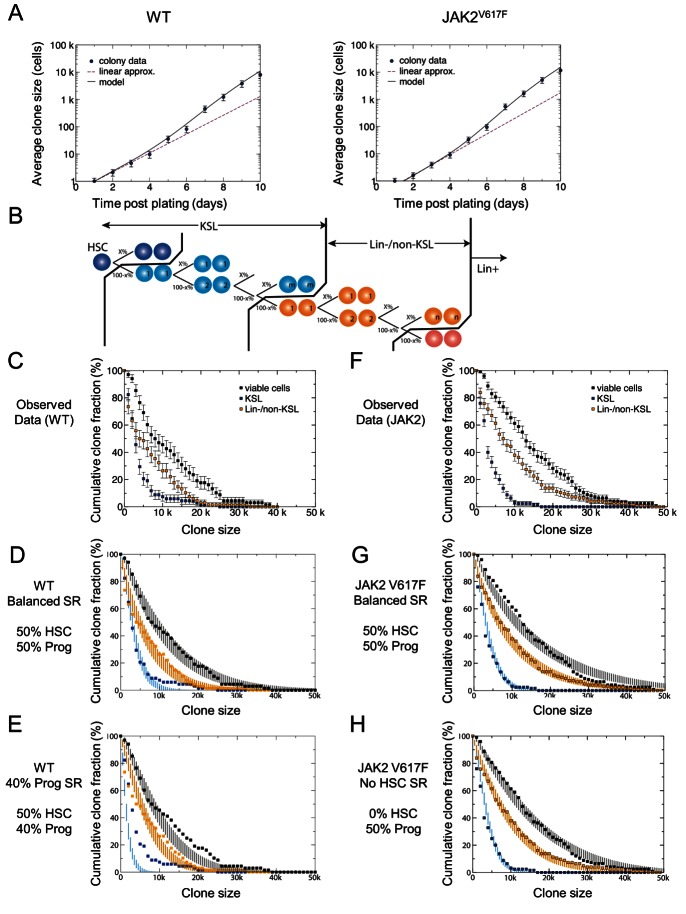
JAK2^V617F^ E-SLAM HSCs, but not progenitors, are tilted toward differentiation. (A) The average clone size data for WT and JAK2^V617F^ E-SLAM HSCs are approximately exponential over the 10-d time course. At early times, the data for both cell types show that the expansion is geometrical, with individual clones expanding from one to two to four to eight cells. After several rounds of division, the average cell division rate appears to accelerate significantly, while nearer to 10 d, there is a deceleration most likely due to cells exiting cycle. The dashed line shows what exponential growth would look like with the average doubling rate of the first 4–5 rounds of division (1.06 for WT and 1.26 for JAK2^V617F^), and the solid line represents the model fit to the actual data points for WT (left) and JAK2^V617F^ (right) clones (for details, see Model S1). Note that, in both cases, division rates must increase to accommodate the expansion measured at day 10. (B) A schematic of the model dynamics. In the WT situation, cells move through a differentiation hierarchy with HSCs at the apex. In the model, the division of an HSC leads to symmetric duplication or differentiation with equal probability (i.e., x = 50%). Cells at the first generation of the differentiating hierarchy then have a capacity to duplicate or symmetrically differentiate into cells in the next tier of the hierarchy, and the model can be tuned to allow x to vary throughout (see Model S1 for further details). (C) The cumulative size distribution of clones 10 d postplating by cell type in WT clones—i.e., the KSL data point at (4k, 40%) in the WT graph—shows that 40% of the colonies have at least 4,000 KSL cells, etc. (D) Comparison of the balanced self-renewal model (i.e., with x = 50% within the entire stem and progenitor cell compartments) with parameters inferred from a fit to the colony growth curve (A) and cell type averages at 10 d postplating, against the experimental data (points) taken from (C) and (E). The vertical lines (color coded by cell type) represent the expected range of fluctuations of the cumulative size distribution due to small number statistics, and are inferred from the average and first standard deviation of the results of the model simulation with 1,000 trials each with a cohort of 68 (WT) and 125 (JAK2^V617F^) colonies, consistent with that used in experiment (for further details and model parameters, see Model S1). (E) The same data set as in 5C and 5D, but with model predictions when just 40% of the progenitor cell progeny (x = 40% for the non-HSCs tiers) remain at the same tier of the hierarchy. Note the departure of the line for the KSL population, which reflects the premature escape of cells from the top of the hierarchy. (F) The cumulative size distribution of colonies 10 d postplating by cell type in JAK2^V617F^ clones. (G) The balanced self-renewal model (i.e., with x = 50% within the entire non-HSC progenitor compartment) overlaid onto the data from JAK2^V617F^ clones with solid lines displaying the predictions of the model. The departure of the model from the observed data is visible in the total viable cells where the model predicts more viable cells in order to produce the observed number of KSL and Lin-/non-KSL cells. (H) Here the lines shown represent a model where HSC self-renewal has been set to 0 implying that every division of an HSC will result in differentiation to the next tier, but progenitor self-renewal remains intact within the rest of the non-HSC progenitor cell compartments. Note the strong overlap of the model with the data points from the JAK2^V617F^ clones. In panels C–H, the total size colony is represented by black, Lin-/non-KSL cells are beige, and KSL cells are blue.

To understand the self-renewal and differentiation capacity of individual HSCs and their progeny, we combined a quantitative analysis of short-term clone size data with a more detailed analysis of the colony size and cell type composition after 10 d in culture using the data presented in Figure 2. Consistent with previous reports [25],[29], HSCs exposed to SCF and IL-11 rarely entered the cell cycle before 24 h and had an average time to first division of approximately 40 h (Figure S1A). After this initial lag, HSC-derived clones underwent steady exponential expansion at a constant rate for the first 4–5 rounds of division, suggesting that few cells, if any, exited the cell cycle (Figure 5A). Clones then underwent a substantial increase in their average cell division rate. At the end of the time course, the average clone size increased less rapidly, consistent with cells committing to terminal differentiation (Model S1 and Figure S2).

To gain further insight into the scale of the lineage hierarchy, and fate behavior of HSCs and their differentiating progeny, we developed a biophysical modeling scheme to address the range of experimental data. On the assumption that HSCs constitute an equipotent pool, we supposed that HSCs and their differentiating progeny are organized in a unidirectional hierarchy. The KSL fraction of mouse bone marrow cell cultures has been shown previously to contain the vast majority of HSCs together with a larger number of progenitor cells [30]. Therefore, we defined KSL as stem and early progenitor cells, Lin^−^/non-KSL as progenitor cells further down the hierarchy, and Lin^+^ as cells that have differentiated (Figure 5B).

Secondly, it was assumed that HSC self-renewal occurs within the culture system. This is supported by transplantation assays that demonstrate the culture conditions used here maintain the input number of stem cells, meaning that cells at the apex of the hierarchy form a self-renewing population [25]. Moreover, transplantation efficiency of single HSC-derived clones (assessed as a binary outcome in primary recipient mice) declines with time in culture [25],[29], suggesting that the balance between HSC differentiation and self-renewal is achieved at the population level, but not at the level of individual cells (i.e., HSCs follow a balanced stochastic cell fate, leading to neutral drift-type dynamics of the clones).

Thirdly, it was supposed that, as HSCs progressively differentiate, they move through a cascade of intermediate tiers, which retain a degree of self-renewal potential before leaving the KSL compartment. On the basis of the colony expansion over the 10-d time course and the average number of KSL cells produced at 10 d postplating, we concluded that approximately seven such distinct differentiation tiers exist in the KSL compartment (Model S1).

Figure 5C shows the cumulative clone sizes derived from the WT data described in Figure 2. Adjusting the division and loss rates to fit the colony growth curve over the 10-d time course (Figure 5A), we find that the model provides a good fit to the cumulative clone size distributions of the KSL, Lin^−^/non-KSL, and total cell population at 10 d postplating (Figure 5C,D, and Figures S3 and S4) if we assume that all proliferative cells undergo perfect (balanced) self-renewal. To assess the quality of the fit, we have used the numerical simulation to estimate the expected range of errors due to small colony number statistics (for details, see Model S1). The robustness of the fit is further reinforced by the relatively poor agreement between the model and experiment even when progenitor cells show only a 10% tilt towards differentiation (compare Figure 5D to Figure 5E). Even with this small bias, the predicted number of KSL cells is shifted to values significantly smaller than that observed in experiment (blue line, Figure 5E). Importantly, these results indicate that balanced self-renewal occurs within multiple tiers of early progenitor cells.

Our transplant data show that long-term self-renewal activity of JAK2^V617F^ HSCs is compromised, and our in vitro studies indicate that JAK2^V617F^ HSCs generate more progenitor cells over the 10-d time course (Figure 2B–D and Figure 3B). We therefore postulated that the self-renewal defect might affect HSCs but not progenitors. Direct comparison of the WT and JAK2^V617F^ colony size data (Figure S3) showed that, although the distributions are significantly tilted toward differentiation, the colonies show the same characteristic dispersion in size and composition. To further explore this possibility, clonal composition data obtained using JAK2^V617F^ E-SLAM HSCs were compared with model simulations in which HSC or progenitor self-renewal was altered. In common with the clones derived from WT HSCs, those created from JAK2^V617F^ HSCs displayed approximately exponential growth (Figure 5A), had a similar delay prior to their first division (Figure S1A), and showed very poor agreement with simulations where progenitor cell self-renewal was abolished or even reduced by only 10% (unpublished data). Furthermore, when the model included perfectly balanced self-renewal (i.e., 50%) of both HSCs and progenitors, the observed fit with data from the JAK2^V617F^ clones was relatively poor, resulting in expected clone sizes much larger than actually observed (Figure 5G and Model S1).

By contrast, when HSCs were endowed with no self-renewal potential (i.e., all HSCs undergo differentiation) but progenitor cell self-renewal remained intact, a good agreement of the model with the experimental data could be obtained (Figure 5H and Model S1). Moreover, satisfactory fits of the model to the data were also found when HSC self-renewal was set at 10% or 20% (Figure S5 and unpublished data). We also undertook the same iterative analysis using old HSCs from WT and JAK2^V617F^ mice, making allowance for their delayed cell cycle entry. The observed cell type distributions agreed well with model predictions for both WT and JAK2^V617F^ clones (Figure S6 and Model S1).

Importantly, this model is not capable of providing a precise prediction of the degree of bias. Nevertheless, taken together, our results suggest that, following the acquisition of the JAK2 mutation, the self-renewal potential of HSCs is diminished while the behavior of their more differentiated progeny is left largely unchanged.

### Paired Daughter Cell Analysis Reveals a Direct Effect by JAK2V617F on HSCs

**Figure 6:**
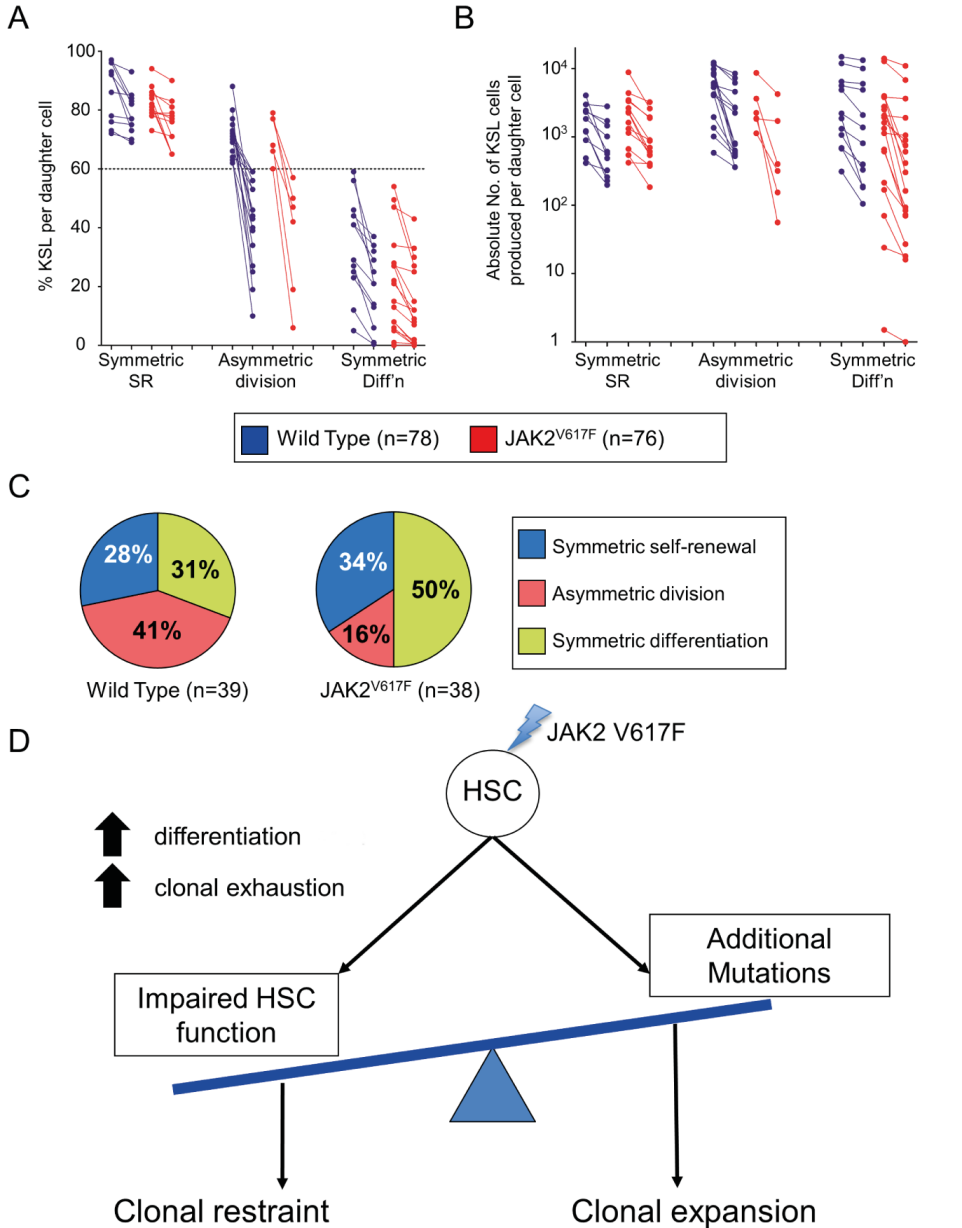
JAK2^V617F^ alters the balance of HSC fate choices. (A) A paired daughter cell analysis of WT and JAK2^V617F^ HSCs shows both daughters differentiate more often from JAK2^V617F^ parent HSCs than from WT HSCs as shown by measuring the percentage of KSL cells remaining after 10 d. Each paired daughter set is connected by a line and the pairs are categorized into symmetric SR (both daughters above the WT average %KSL), asymmetric division (one daughter above and one below the average %KSL), and symmetric differentiation (both daughters below the average %KSL). Note the relative increase in symmetric differentiation at the expense of asymmetric divisions. (B) The same paired daughter pairs are displayed here by the absolute number of KSL cells produced. Here it is clear that some of the JAK2^V617F^ pairs produce very few KSL cells (less than 100 per clone in some of the asymmetric divisions and symmetric differentiation divisions compared to WT HSCs, which are all above 100 KSL cells). (C) The pie graph on the left represents the outcome from 78 WT paired daughters (39 pairs), and the pie on the left represents the outcome from 76 mutant paired daughters (38 pairs). (D) Normally, HSCs will execute one of several programs in concert with the other HSCs to provide the requisite numbers of stem cells, progenitors, and differentiated cells for the organism. JAK2^V617F^ disturbs this balance and increases the likelihood of differentiation. As HSCs with the V617F mutation age, they have both an increased chance of fully exhausting as well as an increased chance of progressing to a more severe disease state, likely due to the acquisition of additional genetic or epigenetic perturbations.

To challenge the prediction that JAK2V617F alters the balance between proliferation and differentiation at the apex of the stem cell hierarchy, we undertook a paired daughter cell analysis to assess the fate outcome of the first division of HSCs from JAK2^V617F^ mice and their littermate controls. To this end, the progeny of the first cell division of input HSCs were split into individual cultures and, after 10 d, assessed by flow cytometry (see Methods S1 for splitting procedure). We elected to use the average fraction of KSL cells from the WT as a benchmark for self-renewal, since HSCs have been shown to undergo approximate balanced self-renewal under these culture conditions (previous transplantation data [20],[25] and Figure 3). We estimated the outcome of the first division based on whether progeny of the doublets were individually above or below the average (see Methods S1 for further detail). The frequency (Figure 6A) and absolute number (Figure 6B) of KSL cells per daughter cell were measured for each doublet to assess the degree of symmetry between daughters. Applying this procedure, the data indicate that the divisions of WT HSCs lead to all three possible fate outcomes in roughly equal proportion (Figure 6C). In particular, divisions leading to symmetric self-renewal appear to be in balance with those leading to symmetric differentiation, as expected. Moreover, when referred to their average KSL content, the data from the JAK2^V617F^ HSCs also showed approximate balance (unpublished data), consistent with balanced self-renewal remaining intact at the lower progenitor tiers, despite compromised HSC self-renewal. Analysis of the fate of JAK2^V617F^ doublets using the average fraction of KSL cells from the WT as the benchmark demonstrated a significant increase in symmetric differentiation divisions (*p* = 0.04), mainly at the expense of fewer asymmetric cell divisions (*p* = 0.01). These data suggest that JAK2V617F directly affects HSC fate choice in vitro, with consequent loss of HSCs.

## Discussion

Establishing and maintaining a clone is a fundamental property of cancers, and it is therefore critical to understand the effect of individual oncogenes on the balance between self-renewal and differentiation. To our knowledge, this study represents the first to isolate single stem cells and study their individual response(s) to a driver mutation associated with a human malignancy. Our results show that JAK2V617F alters HSC fate choices, skewing toward differentiation and proliferation, and quantitative analysis of individual clones predicts that JAK2V617F exclusively affects the self-renewal ability of individual HSCs but leaves intact the expansion capacity of progenitors. This represents a distinct cellular action for JAK2V617F in stem cells compared to progenitor cells, although our in vitro studies do not necessarily imply the same behaviour in vivo and do not address the potential role of the hematopoietic microenvironment. Importantly, the negative effect of JAK2V617F on HSC self-renewal suggests the need for additional mutations to drive clonal expansion consistent with our results showing recovery of HSC self-renewal in transformed animals (Figure 6D).

JAK2^V617F^ mice express a single copy of human JAK2V617F and develop a phenotype that is highly reminiscent of patients with ET. As in JAK2^V617F^ mice, splenomegaly is rare in chronic phase ET patients and the JAK2 mutation is associated with a mild but significant increase in hemoglobin that still lies within the normal range [31]–[33]. The modest increase in platelet counts is also consistent with patient data where the median platelet count is 846 [33], and JAK2V617F-positive individuals with platelet counts in the 400s can readily be identified [34]. Furthermore it is well recognized that a small minority of ET patients transform to PV or MF, consistent with the 10% transformation rate observed in JAK2^V617F^ mice.

It is informative to compare the phenotype of the JAK2^V617F^ mice described here, which express heterozygous human JAK2V617F, with that of other JAK2V617F knock-in models [16]–[18]. Heterozygosity of JAK2V617F is associated with varying degrees of erythrocytosis, an observation that may reflect different gene-targeting strategies or the use of human instead of mouse JAK2V617F (reviewed in Li et al. 2011 [15]). When the stem and progenitor cell compartment was studied, one group described increased numbers of stem/progenitor cells (KSL) in Jak2-mutant compared to WT mice [16], and another reported no difference in either KSL or CD48^−^/CD150^+^KSL frequency or function at 16 wk posttransplantation [17], though a later report has now described a competitive advantage over WT cells that can be observed beyond 1 y transplantation [35]. It is unclear why these models expressing mouse JAK2V617F differ from each other in the timing and magnitude of their effect on HSCs [15],[36]. Importantly, the serial transplantations performed in the Mullally study [17], which follow donor cells through two rounds of transplantation, take place over a total of 3–4 mo compared to the 18 mo that our JAK2^V617F^ cells were followed. Moreover, our experiments use both purified HSC fractions and secondary transplantation analyses to fully characterize stem cell function, and unlike other knock-in models [16]–[18], the function of JAK2 mutant HSCs is compared to WT littermate controls.

Given the phenotypic differences between the various knock-in models, the conclusions of our article relate specifically to the JAK2^V617F^ model studied here, which, as described above, recapitulates many features of human ET. It must also be remembered that HSCs from all of these knock-in models express JAK2V617F at the same time and do not model the acquisition, in a single cell, of a mutation that attains a clonal advantage and drives disease in the presences of nonmutant HSCs. This underscores the usefulness of performing competitive transplantation experiments using purified HSCs (Table 1), where JAK2 mutant HSCs are placed into a wild-type environment alongside wild-type cells.

The stem cell defect that we observe in JAK2^V617F^ mice is also consistent with previous studies of normal individuals and MPN patients. A recent study of nearly 4,000 individuals attending outpatient clinics reported a nearly 1% incidence of JAK2V617F, suggesting that it is insufficient to drive disease [37]. Furthermore, within MPN patients, allele burden is higher in granulocytes compared to CD34^+^ cells [38], only a minority of CD34^+^CD38^−^ stem and progenitor cells bear the JAK2V617F mutation in many individuals with ET or PV [39]–[41], and CD34^+^ cells expressing the mutation failed to out-compete normal cells in transplantation experiments using immunodeficient mice [40],[42]. Moreover, JAK2V617F allele burden is stable over many years in patients with chronic phase ET or PV [43], and the neoplastic clone failed to expand following accidental allogeneic transplantation of donor JAK2V617F mutant cells [44].

Our observations agree with data from patient samples carrying the BCR-ABL mutation. In patients, both JAK2V617F and BCR-ABL are associated with MPNs, are acquired early, and result in expansion of lineage-committed progenitors with overproduction of mature cells. HSCs expressing BCR-ABL are underrepresented in the most primitive cell compartment [45] and display reduced in vitro self-renewal in long-term cultures [46],[47], reduced self-renewal in transplantation experiments in immunodeficient animals [48], and increased genomic instability [49],[50]. Moreover, recent knock-in models of BCR-ABL [51] and FLT3-ITD [52] (a third tyrosine kinase associated with myeloid malignancies) have both been shown to compromise HSC self-renewal in transplantation experiments.

The observation that the JAK2V617F mutation impairs HSC self-renewal needs to be reconciled with its prevalence in MPNs. It is important to note that the defect we observe is relatively subtle and requires serial transplantation assays to be revealed; acquisition of JAK2V617F would therefore not be predicted to result in clonal extinction during the lifespan of a patient. Our results accord with loss-of-function studies using several nontyrosine kinase tumour suppressors (e.g., Rb [53], PTEN [54], p16 [55], and p21 [56]), each of which has been associated with loss of HSC self-renewal, and it has been postulated that tissue stem cells might use a self-renewal disadvantage as barrier to tumor transformation [57]. This concept suggests that subsequent clonal expansion requires a selective advantage, which could reflect acquisition of new genetic lesions, but could also result from an environmental change that selects for mutant HSCs that were previously disfavored [57]. In some MPN patients, mutations in other genes (e.g., TET2) have been shown to precede acquisition of JAK2V617F [58],[59] and may counteract its negative effects on HSC function. Consistent with this concept, TET inactivation results in HSC expansion [58] and the HSC compartment is expanded in some PV patients, in whom the majority of the HSCs do not bear the JAK2 mutation [39]. Such a “pre-JAK2” phase is also consistent with the observation that AML, arising from a JAK2-mutant chronic phase MPN, frequently lacks the JAK2 mutation [43],[60]. Furthermore CD34^+^ cells from patients bearing both TET2 and JAK2 mutations demonstrated robust and increasing chimerism in xenotransplantation experiments, whereas those with a JAK2 mutation alone declined over time [58]. JAK2V617F may play a causal role in acquisition of additional mutations since it is associated with increased DNA damage [19],[61], reduced apoptosis of DNA-damaged cells [62], and as we show in this article, increased proliferation of early progenitors.

It will be important to understand how JAK2V617F cooperates with the increasing number of other lesions being identified in chronic phase MPNs, notably Idh1/2, Tet2, Asxl1, and Dnmt3a (reviewed in [63]). Further characterization of the mechanisms whereby JAK2V617F is associated with subclinical clonal expansions and overt MPNs will illuminate the earliest stages of tumor establishment and subclone competition.

## Material and Methods

### Mice

JAK2^V617F^ mice were generated as described previously [19] and backcrossed onto a C57Bl/6 background for 10 generations. Mice in these studies were between 6 mo and 24 mo post-pIpC injection. The PCR to detect the proportion of recombined allele in HSCs from transformed and recipient animals was performed as described previously (Figure S1F) [19]. All mice were kept in specified pathogen-free conditions, and all procedures performed according to the United Kingdom Home Office regulations.

### Isolation of E-SLAM HSCs

Suspensions of BM cells from adult JAK2^V617F^ or WT mice were isolated from the femurs, tibias, and hips and depleted of red blood cells by a lysis step (BD PharmLyse). E-SLAM cells were isolated as described previously [24] using CD45-FITC [Clone 30-F11 BD Biosciences, San Jose, CA (BD)], EPCR-PE (Clone RMEPCR1560, STEMCELL Technologies, Vancouver (STEMCELL)], CD150-Pacific Blue or PE-Cy7 [Clone TC15-12F12.2, both from Biolegend, San Diego, USA (Biolegend)], and CD48-APC (Clone HM48-1, Biolegend). The cells were sorted on a MoFlo (Beckman Coulter) using the following filter sets [530/30 (for FITC), 580/30 (for PE), 630/40 (for APC), and 450/20 (for Pacific Blue)]. Cells were first sorted at a high rate (10,000–15,000 cells/s) using an EPCR^+^CD48^−^ gate that captured approximately 0.5%–1% of all the viable cells and were then resorted at a slower rate (1–200 cells/s) to improve the efficiency of single-cell sorting. When low numbers of E-SLAM HSCs were required, the single-cell deposition unit of the sorter was used to place 1–10 of these cells into the wells of round-bottom 96-well plates, each well having been preloaded with 50 µL serum-free medium.

### Bone Marrow Transplantation Assays

Donor cells (10^5^ or 10^6^ whole BM) were obtained 6–10 mo after pIpC injection from JAK2^V617F^ mice or WT littermate controls (CD45.2). For purified HSC transplants, 10 E-SLAM HSCs were sorted into 96-well plates as described previously [24]. For transplantations of cells derived from 10-d cultures, 100–400 E-SLAM HSCs were cultured in bulk and various doses transplanted. For secondary transplants, whole BM was obtained and ∼6×10^6^ cells containing a mixture of recipient, competitor, and donor-derived cells were transplanted. All competitor BM cells were obtained from WT C57BL/6J (CD45.1/CD45.2) mice, and between 200,000 and 500,000 whole BM cells were transplanted along with donor cell fractions. For all transplantation assays, recipients were C57BL/6J (CD45.1) mice irradiated with a split dose (2×475 cGy) and all transplants were performed by standard intravenous tail vein injection using a 29.5G insulin syringe. Peripheral blood was collected and analyzed as described previously [64].

### In Vitro Cultures

E-SLAM HSCs were sorted and cultured in serum-free media containing 300 ng/mL SCF and 20 ng/mL IL-11. For the immunophenotyping studies, clones were individually stained and assessed for the expression of Sca1, c-Kit, CD41, CD11b, Ly6g, CD71, and a panel of lineage markers. For assessment of apoptosis, cells were stained with 7-Aminoactinomycin D (7AAD, Invitrogen) and Annexin V FITC (BD). See Methods S1 for clone size calculations and antibody information.

### Paired Daughter Cell Analyses

Single HSCs were isolated and cultured in individual wells of a 96-well plate. At 24 h wells were scored for the presence of a single cell (i.e., any doublets were excluded). At 36 h, wells were again scored for the presence of doublets and any wells with two or more cells were excluded. At 42 h, wells were scored to identify cells that had divided between 36 and 42 h. In order to ensure that all cells were at least 2 h postdivision, these wells were harvested at 44 h, and all contained doublets that had undergone their first division between 36 and 42 h. Daughter cells were separated by harvesting the contents of the entire well and distributing those contents across four newly prepared wells pre-filled with 50 µL of media containing the same amount of SCF and IL-11. Wells that received both daughter cells were excluded from the downstream analysis. Following an additional 8 d of culture (10 d in total), clones were harvested and analyzed individually by flow cytometry.

To assess whether or not an individual clone had differentiated, the average fraction of KSL cells from the WT was used as a benchmark. Each doublet in which the expression levels of both colonies were above the average were scored as a “no differentiation,” while cases with one above and one below were considered to be associated with an asymmetrical fate outcome, etc. While such an assignment for an individual split doublet would be vulnerable to statistical noise due to the stochastic nature of subsequent divisions, we would expect that the average over many doublets would converge onto the true proportions.

### Peripheral Blood Analysis

For all transplantation assays, peripheral blood samples were collected from the tail vein of mice at 4, 8, and 16 wk after transplantation and analyzed for repopulation levels as described previously [64]. Antibodies used were CD45.1-PE (Clone A20, Biolegend), CD45.2-FITC (Clone 104, BD), Ly6g-Pacific Blue (Clone RB6-8C5, Biolegend), Mac1-APC (Clone M1/70, Biolegend), B220-APC (Clone RA3-6B2 eBiosciences, San Diego, CA), and CD3e-Pacific Blue (Clone 500A2, BD).

### Clone Size Calculations and Antibody Information for in Vitro Cultures

At 10 d, clones were estimated to be small (50–5,000 cells), medium (5,000–10,000 cells), or large (10,000 or more cells). No clones had fewer than 50 cells. Ten-day clones were stained with biotinylated lineage marker antibodies (hematopoietic progenitor enrichment cocktail, STEMCELL), c-kit APC (BD), and Sca1-Pacific Blue (Biolegend). To enumerate cells, a defined number of fluorescent beads (Trucount Control Beads, BD) were added to each well and each sample was back calculated to the proportion of the total that were run through the cytometer. Small clones were not able to be assessed individually by flow cytometry and were pooled—in all such cases, the percentage of KSL cells was greater than 90%. For the 14-d immunophenotyping studies, cells were co-stained with CD71-FITC (BD), CD41-PE (BD), Ly6g-Pacific Blue (Biolegend), and CD11b-APC (Biolegend). Flow cytometry was performed on a Cyan ADP (Beckman Coulter) or an LSRII Fortessa (BD) and all data were analyzed using Flowjo (Treestar, USA).

### Assessment of PV and MF Transformation

Blood counts of mice were routinely performed to monitor for disease transformation. Mice were considered to have transformed to PV when hemoglobin levels were greater than 200 g/l. Mice were considered to have transformed to MF when they became cytopenic and had a palpable spleen. Postmortem analysis confirmed transformation by assessing spleen size and histology. Details of each transformed mouse can be found in Table S1.

### Colony Forming Assays

Bulk cultures of 100–400 E-SLAM HSCs were harvested and a proportion of cells were used for colony-forming cell (CFC) assays. Assays were performed in a methylcellulose-based medium (M3434) (STEMCELL) as described by the manufacturer.

### Statistical Analyses

For calculating stem cell frequency and obtaining Chi-squared values, we used the web-based calculator at http://bioinf.wehi.edu.au/software/elda/ [65]. The Fisher Exact test was used to determine whether or not clones from paired daughters had undergone an increased or decreased number of differentiation divisions. For all other *p* values reported, a two-tailed unpaired Student's *t*-test (Microsoft Excel) was used.

## Supporting Information

Figure S1 JAK2^V617F^ E-SLAM HSCs do not enter the cell cycle more quickly than WT HSCs and do not differ in numbers of dead or dying cells in 10-d cultures. (A) A total of 429 E-SLAM HSCs from mice 6–10 mo following pIpC injection (*n* = 251 for JAK2^V617F^, *n* = 178 for wild type) were deposited individually into 96-well plates, visually confirmed to be single cells at 16 h, and then wells were scored every 6–12 h for early time points and once per day from day 5 onward. A cell was scored as having undergone a first division when a second cell could be observed in the well and a second division when a third cell could be seen. A Lowess spline curve was generated in GraphPad Prism (version 4.03) using 248 values estimated based on the marked values in the time course and is shown for each of the first and second divisions of E-SLAM HSCs from each genotype. (B) Representative flow cytomtery plots for cultures of 100–400 E-SLAM HSCs following 10 d of culture in SCF and Il-11. In both the entire pool as well as in the stem/progenitor fraction (Kit+Sca+Lin−, KSL), no differences in 7AAD/Annexin V staining were noted. (C) Individual E-SLAM HSCs were cultured and cell counts were performed on day 2 to determine whether or not they had undergone a division in three independent experiments. No difference was observed between HSCs from wild type (blue bar) and JAK2^V617F^ (red bar) littermates. (D) The bar graph shows the results of cell homing assays that measured the number of HSCs in the BM of recipient mice 36 h after transplantation. No difference was observed in homing efficiency between HSCs from wild type (blue bar) and JAK2^V617F^ (red bar) littermates. (E) The bar graph shows the frequency of E-SLAM HSCs measured in the BM of a single mouse that had transformed to PV 12 mo after pIpC injection. Unlike nontransformed JAK2^V617F^ animals that have reduced E-SLAM numbers, the number of E-SLAM cells was not reduced, but instead appear to be increased compared to an age-matched WT control. HSCs from wild type (blue bar) and JAK2^V617F^ (red bar) are shown.
(TIF)

Figure S2 Expansion of colonies derived from single HSCs over the 10-d time course. Colonies derived from WT (yellow) and JAK2^V617F^ mutant cells (orange) show an approximate exponential increase in size over the 10-d time course. For colonies of less than 50 cells, the total cell number was recorded exactly. Colonies in excess of 50 cells were grouped into three broad categories of small (ca. 300 cells), medium (ca. 2k cells), and large (ca. 10k cells). The logarithmic scale highlights the near-geometric (exponential) expansion of the colonies over the entire 10-d time course.
(TIF)

Figure S3 Direct comparison of WT and JAK2^V617F^ mutant colony size distributions. (A) Data points show the composition of individual colonies derived from WT HSCs (grey) and JAK2^V617F^ mutant cells (yellow) after 10 d. (B) Comparison of the cumulative clone size distribution of colonies derived from single HSCs from WT and JAK2^V617F^ mutants after 10 d. The data suggest that the JAK2^V617F^ mutant data are tilted toward differentiation.
(TIF)

Figure S4 Cell type composition of colonies derived from single HSCs. Data points (yellow) show the composition of individual colonies derived from (A) WT HSCs and (B) JAK2^V617F^ mutant HSCs after 10 d. The grey points are a representative cohort of colonies obtained from the numerical simulation of the model with parameters defined in the main text and Supporting Information. Note that, in both cases, while the numerical simulation captures of the overall shape of the distribution, the scatter of the experimental data is somewhat larger than that predicted by the model dynamics. For further discussion, see the main text and Supporting Information.
(TIF)

Figure S5 Analysis of the degree of bias of JAK2 mutant HSCs toward differentiation. Comparison of the colony growth (left) and cumulative clone size distribution (right), disaggregated by cell type, of the JAK2^V617F^ mutant HSCs with the modeling scheme with a bias of (A) 90% (delta = 0.4) and (B) 70% (delta = 0.2) towards differentiation of the HSC compartment and model parameters defined in the Supporting Information section. Points show the results of experiment. (Error bars denote SEM.) The line on the growth curve shows the model prediction with the given parameters. The bars on the cumulative size distribution show the expected range of statistical fluctuations as predicted by the model dynamics. More precisely, the bars (color coded by cell type) represent the standard deviation of the results of the numerical simulation with multiple trials involving a cohort size of 125 colonies, consistent with that used for the experimental data.
(TIF)

Figure S6 Effect of aging on the colony size distribution. (A) Comparison of the measured size distribution of colonies derived from single HSCs in young (yellow) and old (grey) WT mice after 10 d. Note that, although the average size of the separate compartments in old mice is smaller, the overall distribution is similar in spread and correlation between different cell types. (B) Fit of the measured clone size distribution of the different cell types in colonies derived from single HSCs in WT mice at 10 d postplating (points) with predictions of the model with the parameters the same as that specified in Figure 5A, but with an activation period 0.5 d longer. (Error bars denote SEM.)
(TIF)

Table S1 JAK2^V617F^ animals progress to more severe disease. Each of the mice that progressed to more severe disease is indicated by a unique number (1–10) and its main blood parameters are reported. Hct, hematocrit; Plt, platelets; Hb, hemoglobin; WBCs, white blood cell count; ND, not done. Mice numbered 1 through 6 are from the C57BL/6J background and those numbered 7 through 10 are 129 Sv/C57BL/6J hybrids.
(DOCX)

Methods S1 Description of additional techniques including Isolation of E-SLAM HSCs, Bone marrow transplantation, peripheral blood analysis, clone size, calculations, and antibody information for in vitro cultures, E-SLAM homing assay, and paired daughter cell analyses.
(DOCX)

Model S1 Supplementary theory to explain the mathematical model, its assumptions, and conclusions.
(PDF)

## Abbreviations

BM: bone marrow
CFC: colony forming cell
CFU: colony forming unit
HSC: hematopoietic stem cell
IL-11: interleukin-11
MPN: myeloproliferative neoplasm
PB: peripheral blood
SCF: stem cell factor
WT: wild type
