# Correction: Self-Renewal of Single Mouse Hematopoietic Stem Cells Is Reduced by JAK2V617F Without Compromising Progenitor Cell Expansion

**DOI:** 10.1371/journal.pbio.1002092

**Published:** 2015-03-04

**Authors:** The PLOS Biology Staff

It has come to the authors’ attention that two pieces of information were not included in the Supporting Information. The S1 Fig. legend was missing descriptions of panels F and G. The S1 Methods were missing a description of DNA damage analysis. Both corrected files can be found below.

## Supporting Information

S1 Fig JAK2V617F E-SLAM HSCs do not enter the cell cycle more quickly than WT HSCs and do not differ in numbers of dead or dying cells in 10-d cultures. (A) A total of 429 E-SLAM HSCs from mice 6–10 mo following pIpC injection (n = 251 for JAK2V617F, n = 178 for wild type) were deposited individually into 96-well plates, visually confirmed to be single cells at 16 h, and then wells were scored every 6–12 h for early time points and once per day from day 5 onward. A cell was scored as having undergone a first division when a second cell could be observed in the well and a second division when a third cell could be seen. A Lowess spline curve was generated in GraphPad Prism (version 4.03) using 248 values estimated based on the marked values in the time course and is shown for each of the first and second divisions of E-SLAM HSCs from each genotype. (B) Representative flow cytomtery plots for cultures of 100–400 E-SLAM HSCs following 10 d of culture in SCF and Il-11. In both the entire pool as well as in the stem/progenitor fraction (Kit+Sca+Lin-, KSL), no differences in 7AAD/Annexin V staining were noted. (C) Individual E-SLAM HSCs were cultured and cell counts were performed on day 2 to determine whether or not they had undergone a division in three independent experiments. No difference was observed between HSCs from wild type (blue bar) and JAK2V617F (red bar) littermates. (D) The bar graph shows the results of cell homing assays that measured the number of HSCs in the BM of recipient mice 36 h after transplantation. No difference was observed in homing efficiency between HSCs from wild type (blue bar) and JAK2V617F (red bar) littermates. (E) The bar graph shows the frequency of E-SLAM HSCs measured in the BM of a single mouse that had transformed to PV 12 mo after pIpC injection. Unlike nontransformed JAK2V617F animals that have reduced E-SLAM numbers, the number of E-SLAM cells was not reduced, but instead appear to be increased compared to an age-matched WT control. HSCs from wild type (blue bar) and JAK2V617F (red bar) are shown. F) PCR showing the proportion of recombined allele in E-SLAM HSCs from a mouse that underwent a PV transformation (Trans E-SLAM) and from a primary recipient mouse (Repop E-SLAM). The proportion of recombined allele is also shown in total MNCs from the repopulated mouse (Repop MNC). The control is a mixture of both recombined and floxed alleles. G) Gamma-H2AX foci were enumerated by immunofluorescence in E-SLAM HSCs from 18–24 month old WT and JAK2V617F mice. The proportion of nuclei with 0, 1–5, and more than 5 foci are shown. E-SLAM HSCs from old JAK2V617F mice have fewer HSCs with 0 foci (p = 0.03) and more HSCs with 5 or more foci (p = -0.04).
(TIF)

S1 Methods Description of additional techniques including Isolation of E-SLAM HSCs, Bone marrow transplantation, peripheral blood analysis, clone size, calculations, and antibody information for in vitro cultures, E-SLAM homing assay, and paired daughter cell analyses. (DOCX)
