# Supplementary figures and images for: Self-Renewal of Single Mouse Hematopoietic Stem Cells Is Reduced by JAK2V617F Without Compromising Progenitor Cell Expansion
Source: PLoS Biol. 2013 Jun 4;11(6):e1001576. doi: 10.1371/journal.pbio.1001576 (PMC3672217; doi:10.1371/journal.pbio.1001576)

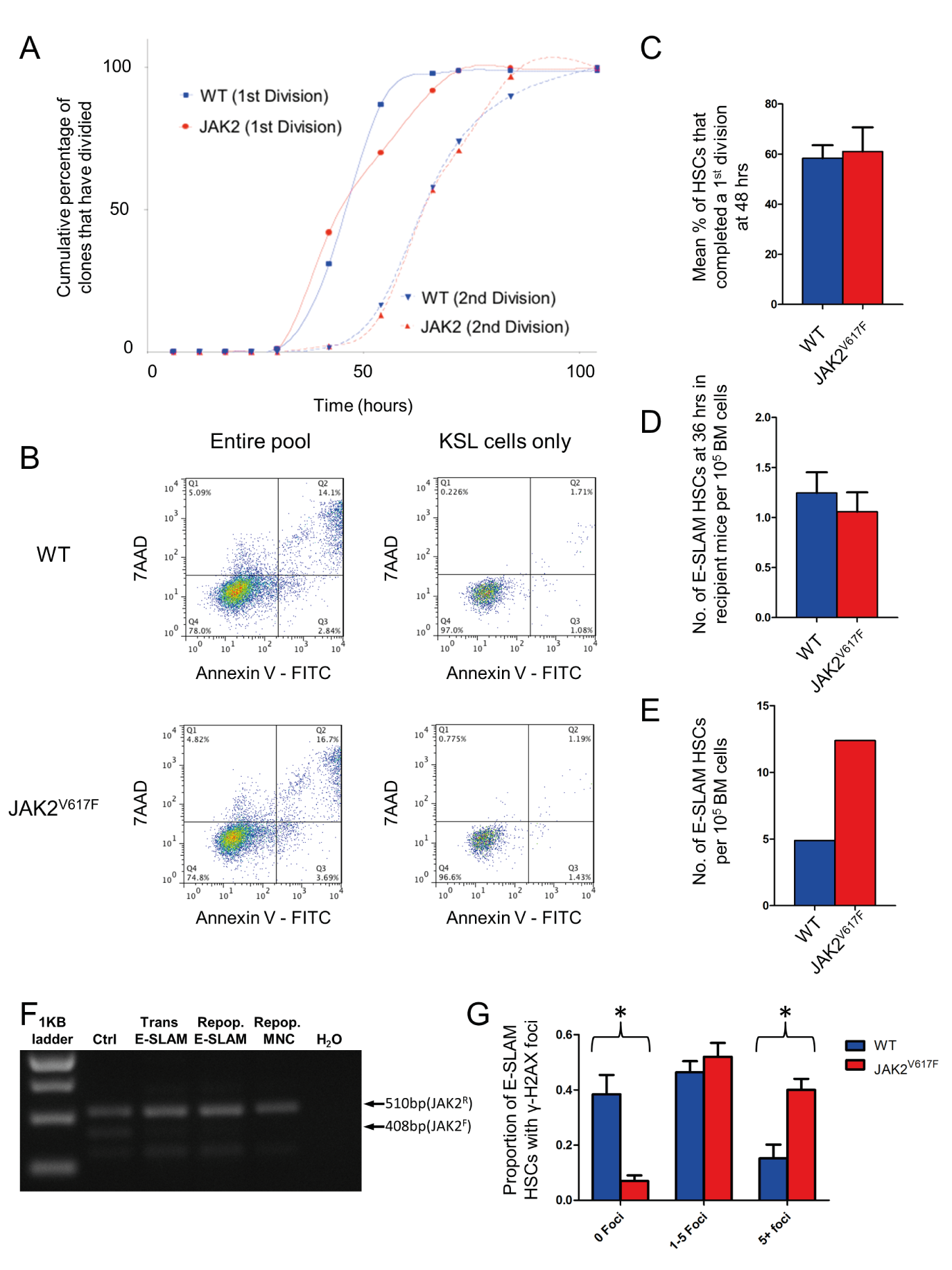

Supplement: Figure S1 — JAK2V617F E-SLAM HSCs do not enter the cell cycle more quickly than WT HSCs and do not differ in numbers of dead or dying cells in 10-d cultures. (A) A total of 429 E-SLAM HSCs from mice 6–10 mo following pIpC injection (n = 251 for JAK2V617F, n = 178 for wild type) were deposited individually into 96-well plates, visually confirmed to be single cells at 16 h, and then wells were scored every 6–12 h for early time points and once per day from day 5 onward. A cell was scored as having undergone a first division when a second cell could be observed in the well and a second division when a third cell could be seen. A Lowess spline curve was generated in GraphPad Prism (version 4.03) using 248 values estimated based on the marked values in the time course and is shown for each of the first and second divisions of E-SLAM HSCs from each genotype. (B) Representative flow cytomtery plots for cultures of 100–400 E-SLAM HSCs following 10 d of culture in SCF and Il-11. In both the entire pool as well as in the stem/progenitor fraction (Kit+Sca+Lin−, KSL), no differences in 7AAD/Annexin V staining were noted. (C) Individual E-SLAM HSCs were cultured and cell counts were performed on day 2 to determine whether or not they had undergone a division in three independent experiments. No difference was observed between HSCs from wild type (blue bar) and JAK2V617F (red bar) littermates. (D) The bar graph shows the results of cell homing assays that measured the number of HSCs in the BM of recipient mice 36 h after transplantation. No difference was observed in homing efficiency between HSCs from wild type (blue bar) and JAK2V617F (red bar) littermates. (E) The bar graph shows the frequency of E-SLAM HSCs measured in the BM of a single mouse that had transformed to PV 12 mo after pIpC injection. Unlike nontransformed JAK2V617F animals that have reduced E-SLAM numbers, the number of E-SLAM cells was not reduced, but instead appear to be increased compared to an age-matched WT control. H [file pbio.1001576.s001.tif]

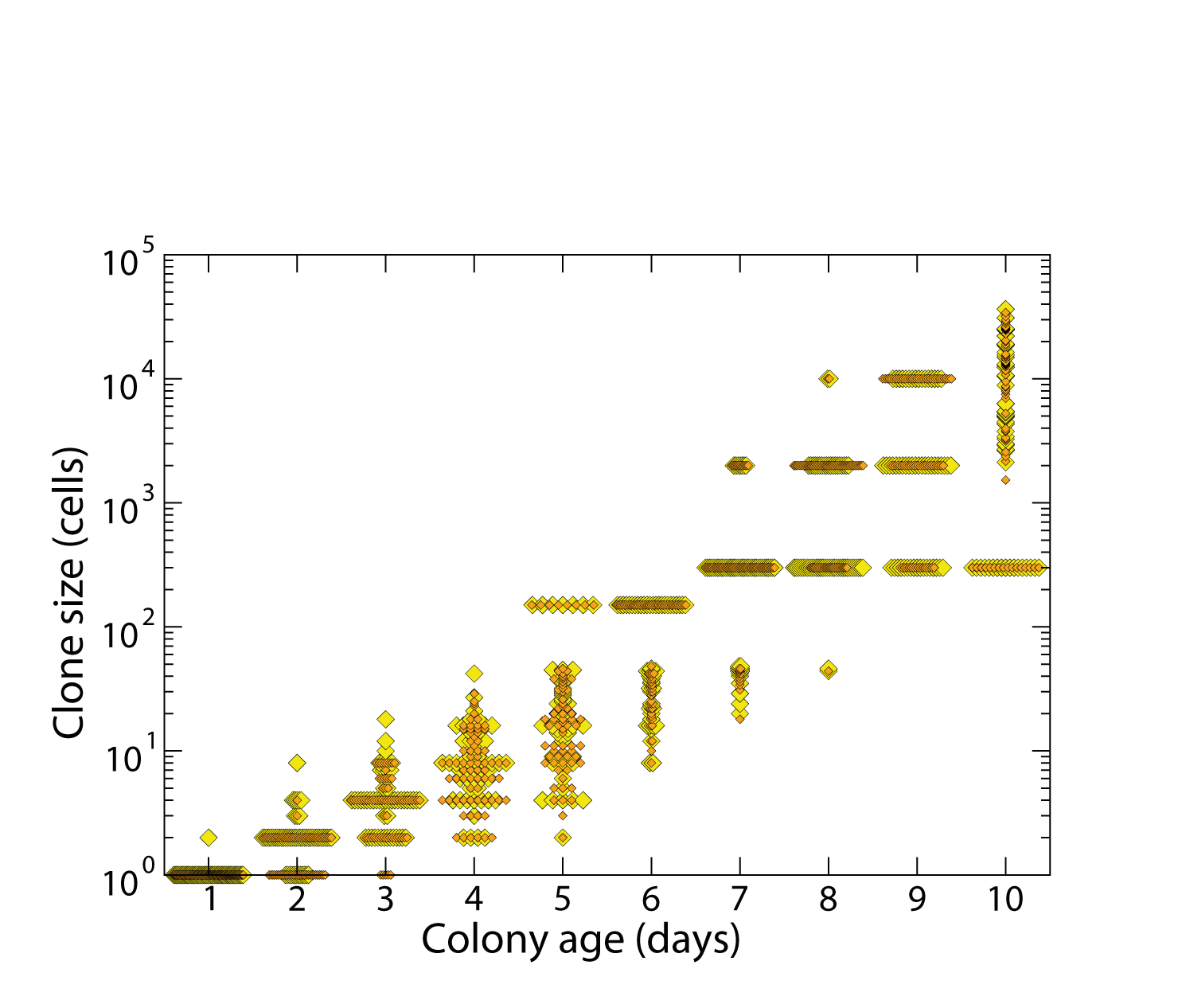

Supplement: Figure S2 — Expansion of colonies derived from single HSCs over the 10-d time course. Colonies derived from WT (yellow) and JAK2V617F mutant cells (orange) show an approximate exponential increase in size over the 10-d time course. For colonies of less than 50 cells, the total cell number was recorded exactly. Colonies in excess of 50 cells were grouped into three broad categories of small (ca. 300 cells), medium (ca. 2k cells), and large (ca. 10k cells). The logarithmic scale highlights the near-geometric (exponential) expansion of the colonies over the entire 10-d time course. (TIF) [file pbio.1001576.s002.tif]

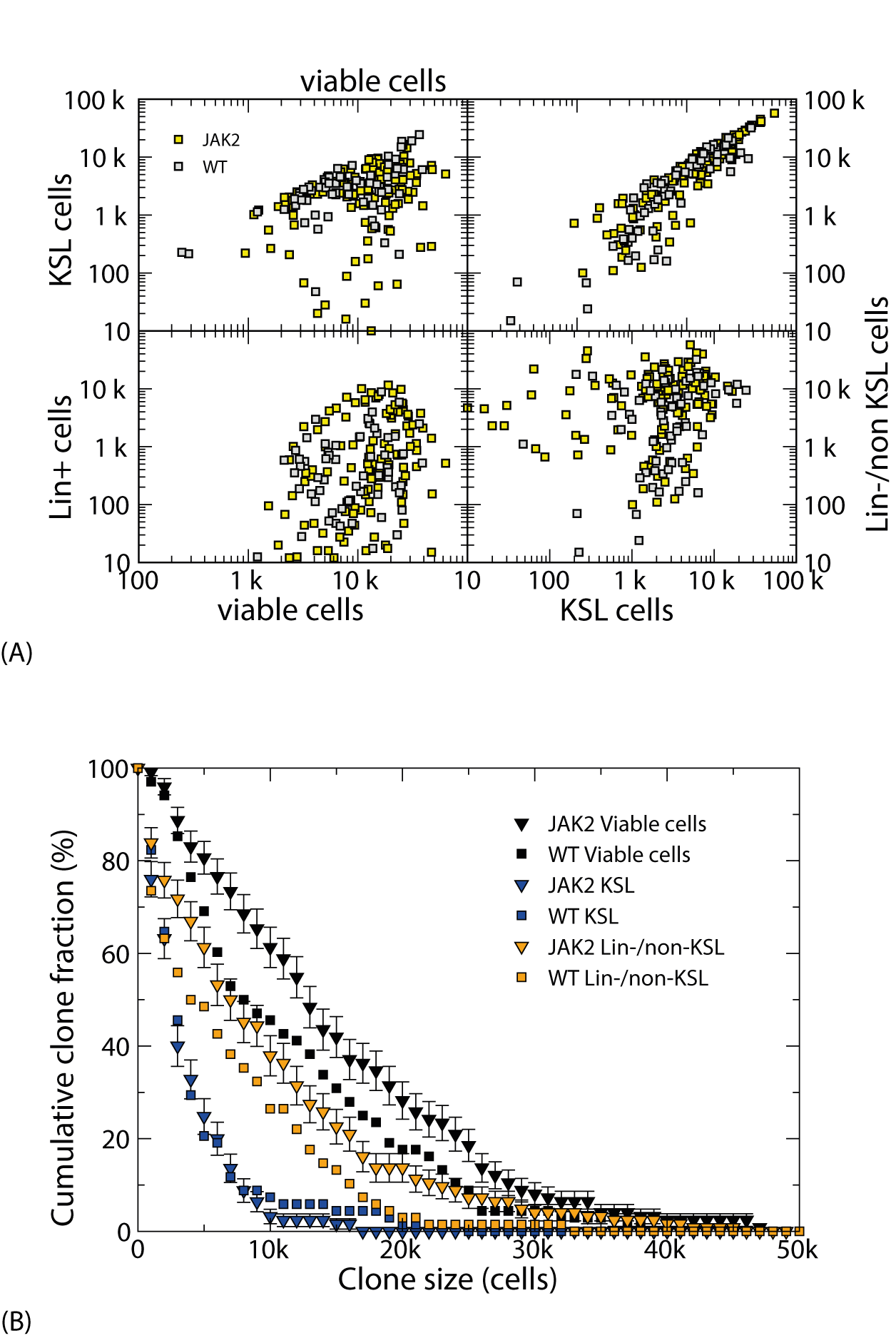

Supplement: Figure S3 — Direct comparison of WT and JAK2V617F mutant colony size distributions. (A) Data points show the composition of individual colonies derived from WT HSCs (grey) and JAK2V617F mutant cells (yellow) after 10 d. (B) Comparison of the cumulative clone size distribution of colonies derived from single HSCs from WT and JAK2V617F mutants after 10 d. The data suggest that the JAK2V617F mutant data are tilted toward differentiation. (TIF) [file pbio.1001576.s003.tif]

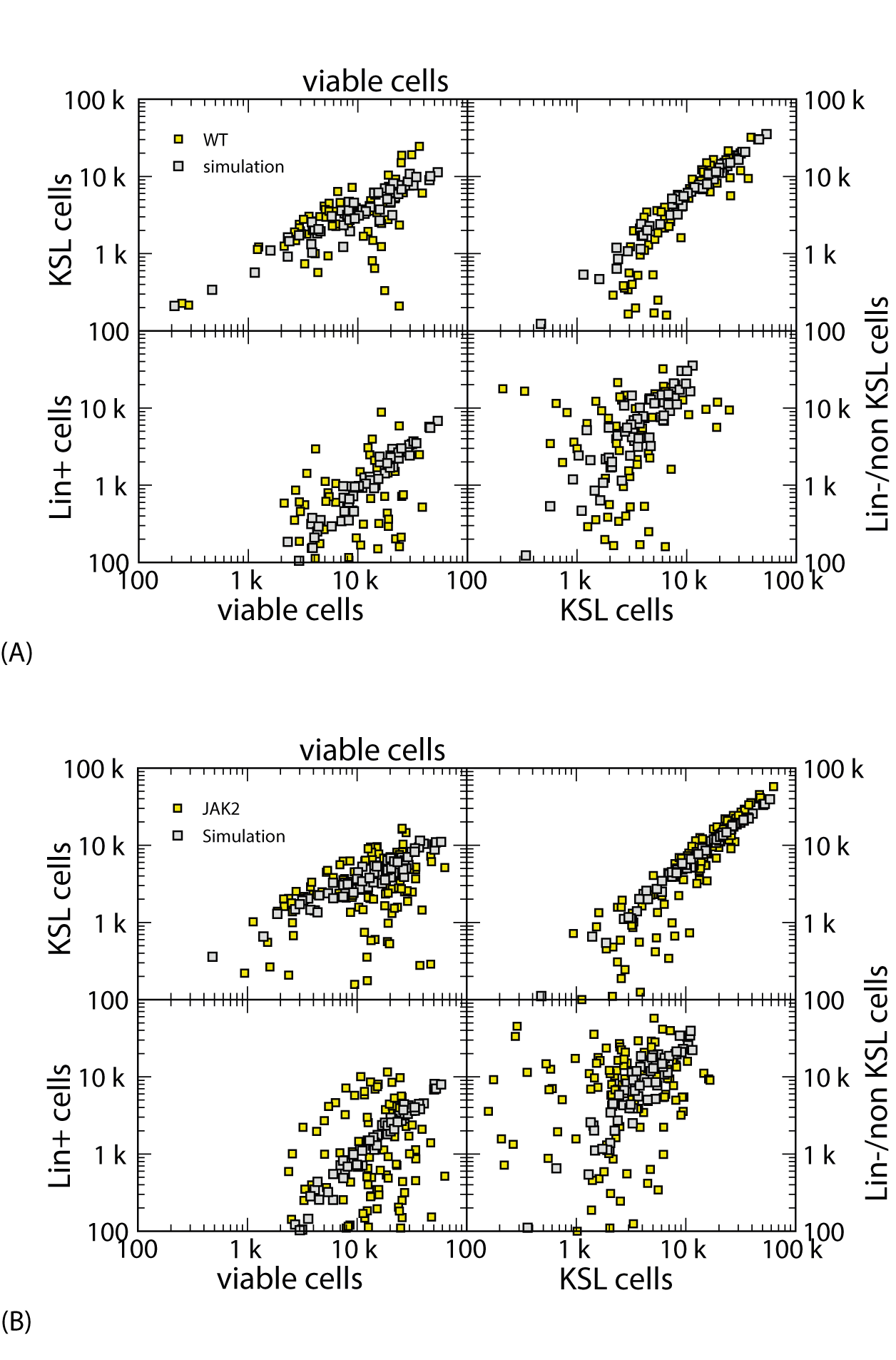

Supplement: Figure S4 — Cell type composition of colonies derived from single HSCs. Data points (yellow) show the composition of individual colonies derived from (A) WT HSCs and (B) JAK2V617F mutant HSCs after 10 d. The grey points are a representative cohort of colonies obtained from the numerical simulation of the model with parameters defined in the main text and Supporting Information. Note that, in both cases, while the numerical simulation captures of the overall shape of the distribution, the scatter of the experimental data is somewhat larger than that predicted by the model dynamics. For further discussion, see the main text and Supporting Information. (TIF) [file pbio.1001576.s004.tif]

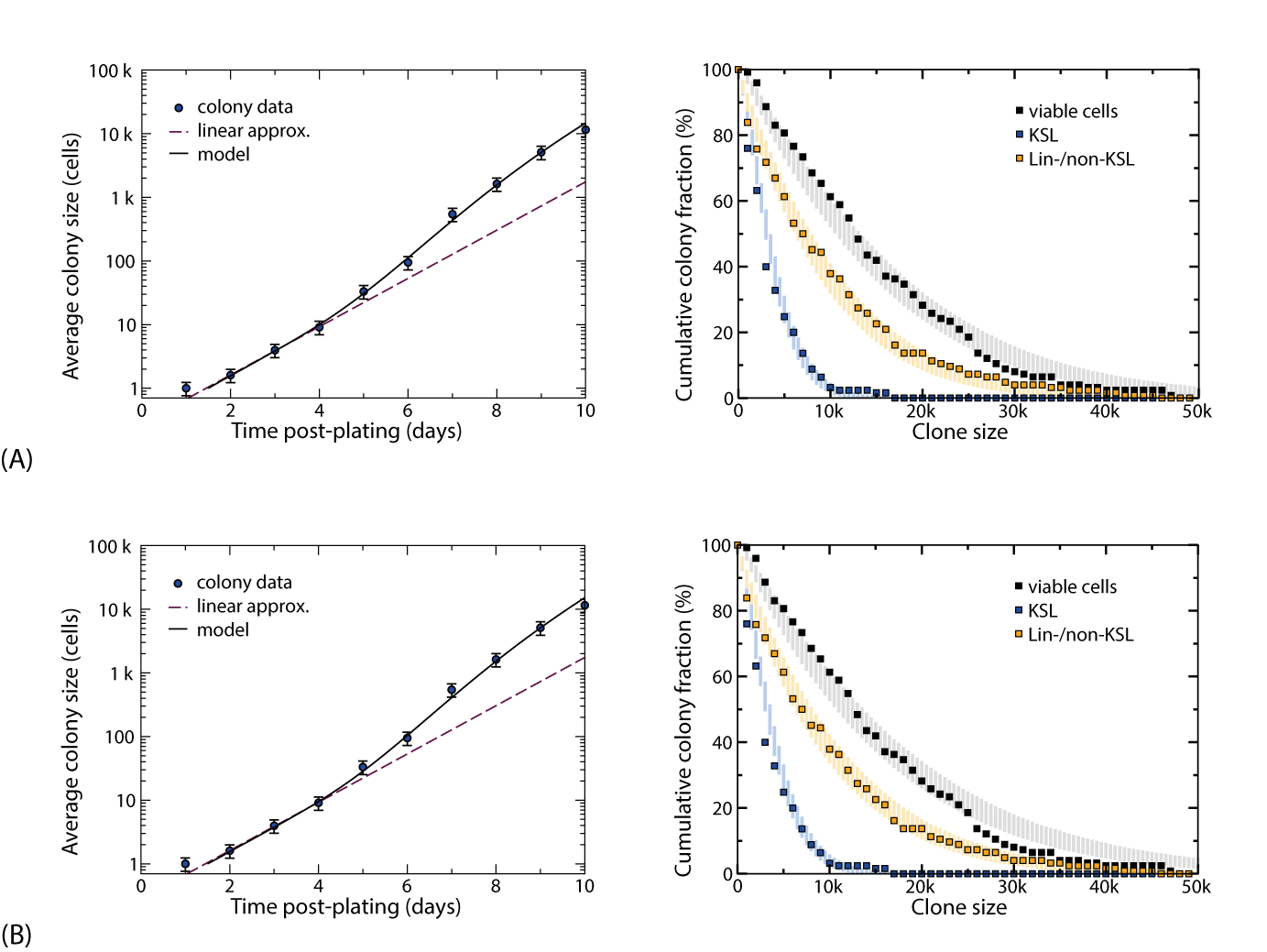

Supplement: Figure S5 — Analysis of the degree of bias of JAK2 mutant HSCs toward differentiation. Comparison of the colony growth (left) and cumulative clone size distribution (right), disaggregated by cell type, of the JAK2V617F mutant HSCs with the modeling scheme with a bias of (A) 90% (delta = 0.4) and (B) 70% (delta = 0.2) towards differentiation of the HSC compartment and model parameters defined in the Supporting Information section. Points show the results of experiment. (Error bars denote SEM.) The line on the growth curve shows the model prediction with the given parameters. The bars on the cumulative size distribution show the expected range of statistical fluctuations as predicted by the model dynamics. More precisely, the bars (color coded by cell type) represent the standard deviation of the results of the numerical simulation with multiple trials involving a cohort size of 125 colonies, consistent with that used for the experimental data. (TIF) [file pbio.1001576.s005.tif]

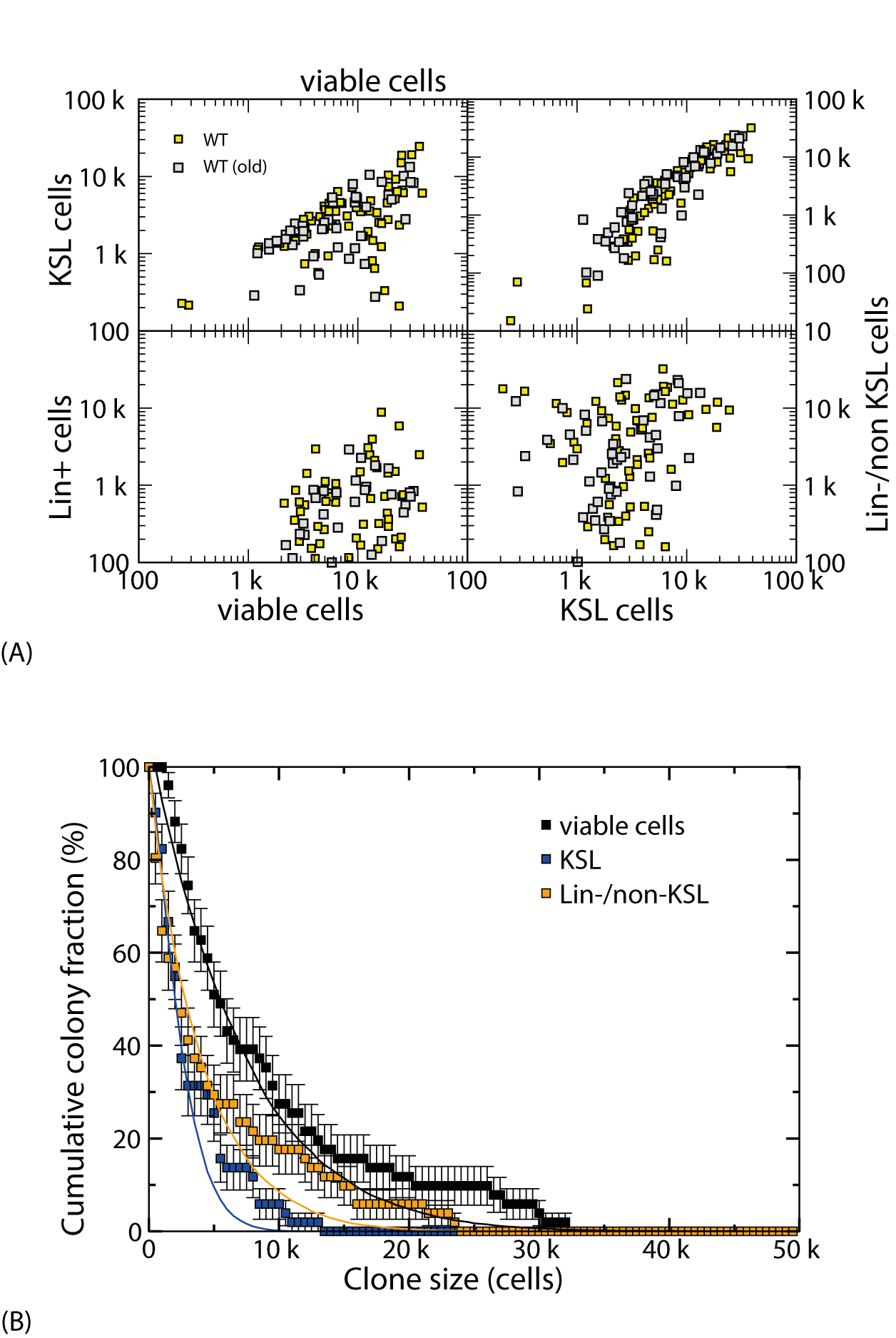

Supplement: Figure S6 — Effect of aging on the colony size distribution. (A) Comparison of the measured size distribution of colonies derived from single HSCs in young (yellow) and old (grey) WT mice after 10 d. Note that, although the average size of the separate compartments in old mice is smaller, the overall distribution is similar in spread and correlation between different cell types. (B) Fit of the measured clone size distribution of the different cell types in colonies derived from single HSCs in WT mice at 10 d postplating (points) with predictions of the model with the parameters the same as that specified in Figure 5A, but with an activation period 0.5 d longer. (Error bars denote SEM.) (TIF) [file pbio.1001576.s006.tif]
