# Supplementary material for: Self-Renewal of Single Mouse Hematopoietic Stem Cells Is Reduced by JAK2V617F Without Compromising Progenitor Cell Expansion
Source: PLoS Biol. 2013 Jun 4;11(6):e1001576. doi: 10.1371/journal.pbio.1001576 (PMC3672217; doi:10.1371/journal.pbio.1001576)
Supplement: Table S1 — JAK2V617F animals progress to more severe disease. Each of the mice that progressed to more severe disease is indicated by a unique number (1–10) and its main blood parameters are reported. Hct, hematocrit; Plt, platelets; Hb, hemoglobin; WBCs, white blood cell count; ND, not done. Mice numbered 1 through 6 are from the C57BL/6J background and those numbered 7 through 10 are 129 Sv/C57BL/6J hybrids. (DOCX) [file pbio.1001576.s007.docx]

| Table S1: JAK2^V617F^ animals progress to more severe disease | | | | | | | | |
| --- | --- | --- | --- | --- | --- | --- | --- | --- |
|  |  |  |  |  |  |  |  |  |
| Mouse Number | Disease Phenotype | Age (# of months  post pIpC) | Hct  (%) | Hb  (g/L) | Plt  (g/L) | WBCs  (x10^6^/mL) | Spleen Weight (g) |  |
| 1 | PV | 5 | 85.8 | 264 | 948 | 9 | 0.41 |  |
| 2 | PV | 6 | 73.6 | 220 | 1133 | 10.9 | 0.916 |  |
| 3 | PV | 12 | 92.2 | 228 | 1166 | 11.7 | 0.306 |  |
| 4 | PV | 12 | 82.8 | 258 | 1043 | 11.2 | 0.275 |  |
| 5 | PV | 4 | 97.2 | 280 | 875 | 11.7 | 0.791 |  |
| 6 | MF | 24 | 12.1 | 92 | 512 | 45.8 | 1.018 |  |
| 7 | PV | 6 | 84.2 | 254 | 913 | 7 | ND |  |
| 8  9  10 | PV  PV  MF | 9  4  19 | 85.2  71.9  33 | 246  212  101 | 1311  922  3106 | 8.6  5.72  3.2 | ND  1.155  1.52 |  |
| Normal |  |  | 51.2-51.8 | 160-164 | 1085-1300 | 10-11 | 0.08-0.081 |  |
