# Supplementary material for: Self-Renewal of Single Mouse Hematopoietic Stem Cells Is Reduced by JAK2V617F Without Compromising Progenitor Cell Expansion
Source: PLoS Biol. 2013 Jun 4;11(6):e1001576. doi: 10.1371/journal.pbio.1001576 (PMC3672217; doi:10.1371/journal.pbio.1001576)
Supplement: Methods S1 — Description of additional techniques including Isolation of E-SLAM HSCs, Bone marrow transplantation, peripheral blood analysis, clone size, calculations, and antibody information for in vitro cultures, E-SLAM homing assay, and paired daughter cell analyses. (DOCX) [file pbio.1001576.s008.docx]

**Supplementary Methods**

*Isolation of E-SLAM HSCs*

E-SLAM HSCs were isolated using CD45-FITC (Clone 30-F11 BD Biosciences, San Jose, CA (BD)), EPCR-PE (Clone RMEPCR1560**,** STEMCELL Technologies, Vancouver (STEMCELL)), CD150-Pacific Blue or PE-Cy7 (Clone TC15-12F12.2, both from Biolegend, San Diego, USA (Biolegend)), and CD48-APC (Clone HM48-1, Biolegend). The cells were sorted on a MoFlo (Beckman Coulter) using the following filter sets (530/30 (for FITC), 580/30 (for PE), 630/40 (for APC), and 450/20 (for Pacific Blue)). Cells were first sorted at a high rate (10,000-15,000 cells/s) using an EPCR^+^CD48^−^ gate that captured approximately 0.5-1% of all the viable cells and were then resorted at a slower rate (1-200 cells/s) to improve the efficiency of single cell sorting. When low numbers of E-SLAM HSCs were required, the single cell deposition unit of the sorter was used to place 1-10 of these cells into the wells of round-bottom 96-well plates, each well having been preloaded with 50 μL SFM.

*Bone Marrow Transplantation*

All competitor BM cells were obtained from WT C57BL/6J (CD45.1/CD45.2) mice and between 200,000-500,000 whole BM cells were transplanted along with donor cell fractions. For all transplantation assays, recipients were C57BL/6J (CD45.1) mice irradiated with a split dose (2 × 475 cGy) and all transplants were performed by standard intravenous tail vein injection using a 29.5G insulin syringe. Peripheral blood was collected and analyzed as described previously^21^

*Peripheral Blood Analysis*

For all transplantation assays, peripheral blood samples were collected from the tail vein of mice at 4, 8, and 16 weeks after transplantation and analyzed for repopulation levels as described previously^21^. Antibodies used were CD45.1-PE (Clone A20, Biolegend) CD45.2-FITC (Clone 104, BD), Ly6g-Pacific Blue (Clone RB6-8C5, Biolegend), Mac1-APC (Clone M1/70, Biolegend), B220-APC (Clone RA3-6B2 eBiosciences, San Diego, CA), and CD3e-Pacific Blue (Clone 500A2, BD).

*Clone size calculations and antibody information for in vitro cultures*

When the clones appeared larger, they were estimated to be small (50-5000 cells), medium (5000-10,000 cells), or large (10,000 or more cells). No clones had fewer than 50 cells.10 day clones were stained with biotinylated lineage marker antibodies (hematopoietic progenitor enrichment cocktail, STEMCELL), c-kit APC (BD), and Sca1-Pacific Blue (Biolegend). To enumerate cells, a defined number of fluorescent beads (Trucount Control Beads, BD) were added to each well and each sample was back-calculated to the proportion of the total that were run through the cytometer. Small clones were not able to be assessed individually by flow cytometry and were pooled – in all such cases, the % of KSL cells was greater then 90%. For the 14-day immunophenotyping studies, cells were co-stained with CD71-FITC (BD), CD41-PE (BD), Ly6g-Pacific Blue (Biolegend), and CD11b-APC (Biolegend). Flow cytometry was performed on a Cyan ADP (Beckman Coulter) or an LSRII Fortessa (BD) and all data were analyzed using Flowjo (Treestar, USA).

*E-SLAM homing assay*

E-SLAM cells (1100) were isolated from 3 JAK2^V617F^ and 3 WT littermates and transplanted into 6 recipient animals after labelling with one of two fluorescent dyes (CellTrace Violet and CellTrace Far Red, Invitrogen). Each dye was first resuspended in 100uL DMSO and the cells were stained at a concentration of 2μM in 1mL of PBS for 30 minutes at 37°C. Following the incubation the cells were equally spread across 6 recipient animals such that each animal received ~180 JAK2^V617F^ and WT E-SLAM cells. For three mice, JAK2^V617F^ E-SLAM HSCs were labelled with the violet dye and WT cells with the Far Red dye and for the other 3 mice the reverse was done to ensure that no bias was introduced by the labelling method. Femurs, tibiae, and pelvic bones were harvested 36 hours post-transplantation and stained for the donor and recipient CD45 molecules before proceeding to flow cytometry analysis. Only cells that were positive for one of the dyes as well as the donor CD45 molecule were considered positive. Approximately 4 million events per recipient were collected by flow cytometry, allowing enumeration of between 20-40 HSCs per sample.

*Paired Daughter Cell Analyses*

Single HSCs were isolated and cultured in individual wells of a 96-well plate. At 24 hours wells were scored for the presence of a single cell (i.e.: any doublets were excluded). At 36 hours, wells were again scored for the presence of doublets and any wells with 2 or more cells were excluded. At 42 hours, wells were scored to identify cells that had divided between 36 and 42 hours. In order to ensure that all cells were at least 2 hours post-division, these wells were harvested at 44 hours and all contained doublets that had undergone their first division between 36 and 42 hours. Daughter cells were separated by harvesting the contents of the entire well and distributing those contents across 4 newly prepared wells pre-filled with 50uL of media containing the same amount of SCF and IL-11. Wells which received both daughter cells were excluded from the downstream analysis. Following an additional 8 days of culture (10 days in total) clones were harvested and analyzed in the same manner as Figure 2.

To assess whether or not an individual clone had differentiated, the average fraction of KSL cells from the WT was used as a benchmark. Each doublet in which the expression levels of both colonies were above the average were scored as a “no differentiation”, while cases with one above and one below were considered to be associated with an asymmetrical fate outcome, etc. While such an assignment for an individual split doublet would be vulnerable to statistical noise due to the stochastic nature of subsequent divisions, we would expect that the average over many doublets would converge onto the true proportions.
