# Supplementary material for: Self-Renewal of Single Mouse Hematopoietic Stem Cells Is Reduced by JAK2V617F Without Compromising Progenitor Cell Expansion
Source: PLoS Biol. 2013 Jun 4;11(6):e1001576. doi: 10.1371/journal.pbio.1001576 (PMC3672217; doi:10.1371/journal.pbio.1001576)
Supplement: Model S1 — Supplementary theory to explain the mathematical model, its assumptions, and conclusions. (PDF) [file pbio.1001576.s009.pdf]

Supplementary Theory

The qualitative behaviour of HSCs derived from the JAK2 V617F knock-in mice in transplantation assays suggests that their long-term self-renewal capacity is diminished in comparison to their WT counterparts. At the same time, when the size and relative contribution of different cell types of colonies derived from JAK2 mutant cells is compared to WT after 10 days post-plating, the differences seem relatively modest given the scale of the expansion. We asked whether the quantitative analysis of the colony data could provide insight into the fate characteristics and proliferative potential of these two HSC populations. By combining a quantitative analysis of short-term colony size data with the longer-term fate characteristics, we will provide additional support to the qualitative conclusions drawn from the results of the transplantation assay. In the following, we will outline the basis and limitations of this simplified modeling scheme.

1 Growth kinetics from the colony assay

When cultured in 96 well plates, CD45+EPCR+CD48-CD150+ cells enriched for HSCs in both WT and the JAK2 mutant divide and expand to form colonies of variable size and cell composition over the 10 day timecourse. While details of the cell composition of individual colonies could only be scored reliably by the 10 day timepoint, the size of colonies could be traced over the whole timecourse from the earliest timepoint (Figures S1,2). Analysis of the average size of the statistical ensemble of colonies, shown in Figure 5A, reveals a steady, geometric (i.e. exponential), expansion of both WT and JAK2 colonies over the first few rounds of division, following a short "activation" period after plating (Figures S1 and S2). During this early phase of growth, we estimate a cell doubling rate for WT cells of approximately once per day, with an activation period of around a day. For the JAK2 mutants, the cell doubling rate appears slightly faster, but still comparable to WT, with an activation period of a little longer at around 1.5 days. However, the small degree of variability of the activation time between different experiments (data not shown) suggest that the difference between WT and JAK2 mutant cells is not statistically significant. This geometric expansion

persists at a constant rate for some 3-5 rounds of division after which the data show a significant and relatively abrupt increase in the average cell division rate over 5-6 further rounds of division. At later timepoints, the average clone size increases less rapidly as cells begin to commit to terminal differentiation.

To gain further insight into the differentiation pathways, and pattern of cell fate choice, we can make use of the longer-term 10 day data, where the individual colony composition can be assessed according to expression of markers of the undifferentiated cell compartment (viz. KSL expression), progenitor cell compartment (Lin^-/non KSL), and differentiated cells (Lin^+). From the raw data, several qualitative features emerge. Referring to Figures S3A,B, we can find WT colonies with as many as 40k cells, while other colonies contain fewer than 1k, a separation of some 5 generations of cell division. If we suppose that such large colonies arise from geometric expansion, we are led to conclude that cells have undergone as many as $\ln 14,000/\ln 2 \simeq 15$ rounds of division. Even if we take the average colony size of 9k cells, we estimate that cells have undergone some 13 rounds of division over the 10 day timecourse. With the measured activation period from the short-term dynamics, this translates to an average cell division rate of ca. $13/9 = 1.4$ per day over the measured timecourse. Moreover, since the early undifferentiated progeny cycle at a lower rate, we can conclude that cells further along the differentiation pathway must cycle at a rate that is in excess of this figure, as indeed suggested by the average colony growth (Figure 5A).

From these findings, several important conclusions follow. The persistent geometric increase in colony size shows that, at least up until day 10, relatively few cells have exited cell cycle. This observation is consistent with the limited expression of lineage markers in colonies at day 10. Since the majority of cells have undergone some 13 rounds of division, this suggests that, either the differentiation cascade has a very large number of compartments, or the progenitors in the cascade retain a significant self-renewal potential which inhibits the progression of cells through the hierarchy. The latter scenario is supported by the relative distribution of different cell types which appears broad at day 10 (Figures S3A,B). To make further progress it is useful to turn to a more quantitative analysis of the colony data.

2 Modeling the differentiation cascade of HSCs in culture

To analyze the clone fate measurements, our strategy will be to develop the simplest modeling scheme that is consistent with the data. However, our goal is not to define in detail

every element of the differentiation heirarchy. Rather, we aim to use the model to assess the approximate self-renewal potential of HSCs and their differentiating progeny, and to use this information to develop further experimental assays to challenge the model.

To develop this program, we will assume that HSCs in both the WT and the JAK2 mutant represent functionally homogeneous populations in which their respective proliferative and differentiation capacity are statistically equivalent, i.e. the colony forming capacity and lineage potential of individual HSCs is determined simply by chance fate decisions. Support for this central assumption will be provided through the success of the scheme in describing the range and complexity of the clonal fate behaviors. Second, we will suppose that HSCs and their differentiating progeny are organized in a unidirectional hierarchy. We refer to cells at the apex of the hierarchy as type S* (for stem cell). Cells lower in the hierarchy but still belonging to the KSL population will be denoted as type S (reflecting a degree of stem cell potential), cells negative for lineage marker expression but not in the KSL population are denoted as type P (for progenitor cell), and cells positive for lineage markers by D (for differentiated). Of course, in the biological system, commitment may not be irreversible and cells may transfer reversibly between different cell compartments. However, provided the frequency of such events is small, they can be safely neglected in the current modeling scheme.

In previous studies and this work, evidence from serial transplantation assays have shown that WT cells at the apex of the cellular hierarchy, S*, form a self-renewing population, even under culture conditions. At the same time, we know that the transplantation efficiency of single-HSC derived colonies tends to diminish over time, with approximately 30% of colonies able to engraft irradiated transplantation recepients long-term after 4 days in culture. The ability to combine long-term self-renewal capacity with differentiation suggests that, under culture conditions, HSCs follow a pattern of balanced stochastic cell fate characterized by the process,

$$\mathrm{S}^* \overset{\lambda}{\mapsto} \begin{cases} \mathrm{S}^* + \mathrm{S}^* & \mathrm{Pr.}\ r \\ \mathrm{S}^* + \mathrm{S}_1 & \mathrm{Pr.}\ 1-2r \\ \mathrm{S}_1 + \mathrm{S}_1 & \mathrm{Pr.}\ r \end{cases} .$$

Here λ denotes the cell division rate, and the parameter r controls the balance between symmetric and asymmetric cell division. More precisely, following cell division, with probability $1-2r$, the daughters adopt asymmetric fate with one cell remaining in the HSC compartment, S*, and other moving to the first generation of cells that are committed to a differentiation

pathway, denoted as S_1. Then, with probability r, cells either undergo symmetric duplication, or symmetric differentiation,

If we focus on the HSC population alone then, by construction of the model, the total number of HSCs in a large colony assay will remain approximately constant over time, but they will become increasingly invested in a diminishing fraction of surviving colonies, i.e. according to this stochastic dynamics, the HSC content of the colonies would be predicted to follow a "neutral drift" type process. Such behaviour is not unprecedented: a similar pattern of behaviour has been reported in keratinocyte progenitors both in vivo and under culture conditions (Clayton et al., 2007). Indeed, if we take into account the transplantation efficiency of the 4 day colony data together with the inferred HSC division rate, we would conclude that the parameter r is large and close to its maximum value of $1/2$. For simplicity, in the following, we will set $r = 1/2$, after which the dynamics takes the somewhat simpler form,

$$\mathrm{S}^* \stackrel{\lambda}{\mapsto} \begin{cases} \mathrm{S}^* + \mathrm{S}^* & \mathrm{Pr.}\ 1/2 \\ \mathrm{S}_1 + \mathrm{S}_1 & \mathrm{Pr.}\ 1/2 \end{cases} .$$

As cells progressively differentiate, we will suppose that they move through a cascade in which the intermediate tiers retain some self-renewal potential. Once again, for simplicity, we will characterize the cell fate kinetics by limiting attention to symmetrical fate outcome leading to duplication or differentiation further down the hierarchy, neglecting the potential for asymmetrical fate outcome. More precisely, we will suppose that differentiating cells in the KSL compartment are defined by the hierarchy,

$$\mathrm{S}_n \stackrel{\lambda_n}{\mapsto} \begin{cases} \mathrm{S}_n + \mathrm{S}_n & \mathrm{Pr.}\ 1/2 - \Delta_n \\ \mathrm{S}_{n+1} + \mathrm{S}_{n+1} & \mathrm{Pr.}\ 1/2 + \Delta_n \end{cases} ,$$

where S_n represent cells expressing KSL markers in generation n of the hierarchy, λ_n denotes the corresponding cell division rate, and Δ_n controls the degree of bias towards differentiation. Cells of type $\mathrm{S}_{n=1}$ are generated through the differentiation of HSCs, which in turn give rise to cells of type $\mathrm{S}_{n=2}$, and so on. In the particular case that all cell divisions lead to differentiation, i.e. $\Delta_n = 1/2$, the model coincides with that recently considered by Haeno et al. (Haeno et al., 2009).

After several rounds of amplification, we suppose that cells in generation M, S_M, give

rise to progenitors in which the expression of c-Kit and Sca1 is down-regulated,

$$\mathrm{S}_M \overset{\lambda_M}{\mapsto} \begin{cases} \mathrm{S}_M + \mathrm{S}_M & \mathrm{Pr.}\ 1/2 - \Delta_M \\ \mathrm{P}_1 + \mathrm{P}_1 & \mathrm{Pr.}\ 1/2 + \Delta_M \end{cases} .$$

Once again, we will assume that the Lin^-/non KSL progenitor cell compartment is organised in a short hierarchy with component cells retaining a degree of self-renewal potential, setting

$$\mathrm{P}_n \overset{\kappa_n}{\mapsto} \begin{cases} \mathrm{P}_n + \mathrm{P}_n & \mathrm{Pr.}\ 1/2 - \delta_n \\ \mathrm{P}_{n+1} + \mathrm{P}_{n+1} & \mathrm{Pr.}\ 1/2 + \delta_n \end{cases} ,$$

where κ_n represent the corresponding cell division rates and δ_n the potential degree of imbalance. Then, after N tiers of amplification, we suppose that this compartment commits to terminal differentiation, upregulating expression of lineage markers,

$$\mathrm{P}_N \overset{\kappa_N}{\mapsto} \begin{cases} \mathrm{P}_N + \mathrm{P}_N & \mathrm{Pr.}\ 1/2 - \delta_N \\ \mathrm{D} + \mathrm{D} & \mathrm{Pr.}\ 1/2 + \delta_N \end{cases} .$$

Finally, in response to culture conditions, we suppose that Lin^+ cells undergo cell death at rate Γ. At the same time, we suppose that cell death in the proliferative cell compartments makes a minimal contribution and can, therefore, be neglected. Altogether, the model dynamics is summarized by the schematic in Figure 5B.

Even with this level of complexity, one may be reasonably concerned that this model represents a gross oversimplification of the true cell dynamics. As well as neglecting the potential for asymmetrical fate outcome or reversible transfer through the hierarchy, the model further assumes that differentiation occurs in a sequence of step-like processes between neighbouring tiers in the hierarchy. Further, at each level of the hierarchy, the model assumes that cells in each tier behave as an equipotent pool, with their fate characteristics not influenced by local factors from the environment or signals from neighboring cells. However, as mentioned before, our aim here is not to develop a detailed biophysical modeling scheme – with so many adjustable parameters, such an enterprise would, in any case, be misguided. Rather, our goal is to establish whether the principle aspects of the cell fate choice and kinetics can be captured by a simple modeling scheme that can discriminate the behavior of WT and JAK2 mutant cells, and can be challenged by further experimental measures. Therefore, instead of introducing further detailed refinements of the model, we will implement additional simplifications to constrain the modeling scheme still further.

In the following, we will begin by focusing on WT cells turning later to address the in vitro behavior of the JAK2 mutant HSCs. For WT cells, we will begin by supposing that, in common with the HSCs, the self-renewal capacity of cells in each tier of the hierarchy is *perfectly balanced*, setting $\Delta_n = \delta_n \equiv \Delta = 0$. Later we will challenge the validity of this assumption by optimizing the fit of the data to changing imbalance. In this approximation, let us consider the predicted dynamics of the constituent populations. If we assume that the times between consecutive HSC divisions are random and uncorrelated, drawn from an exponential (i.e. Poisson) distribution with an average division rate λ, at the population level, the average number of HSCs (per colony) stays fixed at $n_{\rm S} = 1$, while the average number of $\rm S_1$ cells is specified by the kinetic equation $\dot{n}_{\rm S_0} = \lambda n_{\rm S}$, i.e. at rate λ, HSCs give rise to one $\rm S_1$ cell on average. Integrating, we therefore have that $n_{\rm S_1}(t) = \lambda t$, where t denotes the time post-plating. Once again, although the average is predicted to grow linearly with time, by the nature of the stochastic dynamics, $\rm S_1$ type cells will not be evenly distributed between different colonies.

At the next tier in the hierarchy, we have $\dot{n}_{\rm S_2} = \lambda_1 n_{\rm S_1}$, leading to the average $n_{\rm S_2}(t) = \frac{1}{2}(\lambda t)(\lambda_1 t)$. Iterating this procedure, we thus obtain as the total average number of cells in the KSL compartment, $n_{\rm KSL} = 1 + \sum_{n=1}^{M} \frac{1}{n!} \prod_{m=0}^{n-1}(\lambda_m t)$, with $\lambda_0 \equiv \lambda$. Indeed, if M were infinite, and λ_m all equal, this would sum to an exponential, as expected for a purely geometric branching-type process. In practice, with the KSL compartment terminated at a fixed generation M, the geometric nature of the expansion means that the sum will be effectively dominated by this outermost tier.

From the experimental data, we find that, at 10 days post-plating, the average colony size for WT HSCs is around 11k cells (Figure 5A). Since the vast majority of these cells are still in cycle, we can use this figure to obtain an estimate of the average number of rounds of division assuming that division follows a Poisson (as opposed to synchronous) process, i.e. $n_{\rm rounds} \simeq \ln(11,000) \simeq 9$. Then, if we assume that the division rate at the latter stages of the hierarchy are roughly constant, as implied by the geometric pattern of growth at later times (Figure 5A), and set by λ_M, we can estimate the size of the KSL population as approximately $\sum_{n=0}^{M}(\lambda_N t)^n/n!$. When compared to the measured average of $n_{\rm KSL} \simeq 4\text{k}$ cells per colony, for $\lambda_M t \simeq n_{\rm round}$, this equates to $M \simeq 7$. This means that, immediately below the HSCs, KSLs involve some 7 layers in the hierarchy. Similarly, if we focus on the progenitor cell compartment, following a similar line of reasoning, from the measured Lin$^-$/non-KSL average of 6.5k cells, we estimate approximately $N = 2$ rounds in its hierarchy.

Finally, to complete the specification of the model dynamics, we must define the relative rates of cell division for the different compartments. From the short-term data, it is evident that the first 5-6 rounds of division occur at a constant and slower rate than the subseuqent rounds of division. After that, the division rate is approximately doubled. We will therefore set $\lambda_{n\leq 5} = \lambda$, while $\lambda_{n>5} = \kappa_n = \kappa \sim 2\lambda$.

3 Numerical simulation

3.1 WT HSC-derived colonies

Although the model dynamics of the HSCs and their progeny is comparatively straightforward, defined by just the two division rates, λ and κ, and loss rate Γ, the time-evolution of the size distribution cannot be obtained analytically. Moreover, with so many elements in the hierarchy, even a numerical integration of the corresponding Master equation for the joint probability distribution seems unfeasible. However, the model is straightforward to simulate computationally. It is the results of these numerical simulations that we use to fit and analyze the range of experimental data.

Alongside the 10 day timecourse recording the average growth dependence of the colonies, to properly challenge the model, it is important to explore the detailed size distribution of cell types at the 10 day timepoint. Since the FACS measurements could only be reliably completed on colonies in excess of 500 cells, the analysis of the 10 day timepoint is focused on colonies in excess of this size. To fit the data, we adjust the three parameters, λ, κ and Γ to match the growth of the average colony size over the 10 days timecourse (Figure 5A), using the measured time-offset of one day associated with the activation of cells (Figure S1A). With these three parameters fixed, we then examine whether the model yields the correct form of the size distributions, disaggregated by cell type (Figure S3B).

In carrying out the fitting procedure, we note that the overall cell division rate of the most undifferentiated cells, λ, is heavily constrained by the timecourse data. With $\kappa \simeq 2\lambda$ also constrained, only the loss rate Γ can be freely adjusted to fit the data. Implementing this program for WT cells, we obtain the best fit of the model to the measured growth dynamics over the 10 day timecourse for $\lambda = 0.95 \pm 0.05$ per day, $\kappa = 1.64 \pm 0.05$ per day, and $\Gamma = 1.8 \pm 0.1$ per day (Figure 5A). Then, with these values of the parameters, the measured cumulative clone size distribution, defined as the chance of finding a clone with more than n

cells of the given type, as assayed by marker expression at 10 days post-plating, shows the same characteristic broad (exponential-like) distribution, with the correct partitioning of the relative populations (Figures 5C,D).

Although, when referred to the joint scatter of individual colony sizes, disaggregated by cell type (Figure S4A), the overall pattern of behavior of experiment and numerical simulation is similar, with entries showing a reasonable overlap, the data from the simplified theoretical model appear slightly more clustered than the experimental cohort, particularly in the correlation between the total colony size (termed viable), and the differentiated (Lin^+) cell population as well as the correlation between the KSL cells and the Lin^-/non-KSL cells. Indeed, the former may simply reflect the limitations of the "linear" hierarchy that does not allow cells to exit cell cycle from less differentiated cell types. Equally, it may be a signature of variability in the survival probability of different Lin^+ cells, features that lie outside the scope of the simplified modeling scheme considered here.

Before turning to the behaviour of the JAK2 mutant cells, we must first address the quality of the fits and the validity of the modeling scheme. In particular, the analysis of the data relied upon the somewhat extreme assumption that all tiers of the hierarchy had the same – perfect – self-renewal potential. What happens if we allow progenitor cells to become biased towards differentiation? Once again, taking the imbalance to be the same for all tiers in the hierarchy, $\Delta_n = \delta_n \equiv \Delta$, if we set $\Delta = 1/2$, consistent with the model considered by Haeno et al. (Haeno et al. 2009), we can find no range or parameters (at least within the scope of this very simplified modeling scheme) that can be made consistent with the experimental data. In short, to engineer conditions with consistent numbers of KSL cells, vast numbers of cells must have progressed down the differentiation pathway leading to unreasonably large numbers of Lin^-/non-KSL and Lin^+ cells. But what happens if we adjust Δ to some intermediate value?

To assess the quality of the fit, we must introduce a means to quantify errors. In reference to the clone size distributions at 10 days post-plating, the main source of experimental uncertainty is associated with small number statistics. For the WT HSCs, the cumulative clonal size data in Figure 5C is derived from some 68 colonies. The statistics of the largest clone sizes (top 5%) will therefore be dominated by just a few rare events, where the simplest regression-type analysis is questionable. Therefore, to properly assess the statistical significance of the analysis, we have used the modelling scheme itself to estimate the expected range of experimental variability. More precisely, we have used the model to recover the expected statistical variation in the cumulative clone size distributions by examining at

the results of multiple simulations (1000 trials) each involving a cohort of 68 colonies (in the case of WT cells). We have then represented the expected range of variability by the standard deviation in the numerical results for each data point. When the data can be accommodated within the first standard deviation, we have declared the fit to be satisfactory while, when the data lies outside one standard deviation for some or all of the data points, we consider the agreement poor.

On this basis, the agreement between the balanced model (with $\Delta = 0$) and experiment is good (Figure 5C,D). However, Referring to Figure 5E, even for a relatively small bias towards differentiation with $\Delta = 0.1$, the optimal fit of the model to the colony growth curve (with $\lambda = 0.97$ per day, $\kappa = 1.65$ per day, and $\Gamma = 1.8$ per day) translates to a relatively poor agreement of the cumulative clone size distributions for the different cell types. In particular, although we can tune the overall average number of viable cells and, to some extent, the distribution to approximately the correct form at 10 days post-plating, the distribution of KSL cells is poorly reproduced. As expected, the tilt towards differentiation allows these cells to rapidly escape the KSL pool and enter the differentiating phase. We therefore contend that, within the scope of the present simplified modeling scheme – with all of its stated limitations – a model of perfect self-renewal provides a better match to the range of experimental data.

3.2 JAK2 mutant HSC-derived colonies

With this background, we now turn to the study of the JAK2 mutant. First, although at 10 days post-plating, the WT and JAK2 mutant cells show significant differences in the average colony size and composition (Figure 2), analysis of the joint distribution of cell types (Figure S3A) and the cumulative clone size distribution (Figure S3B) show that these differences are relatively subtle. In both cases, the data show a broad distribution of colony sizes with significant overlaps between the WT and JAK2 colonies (Figure S3A). However, when these datasets are projected onto the cumulative distributions, it is apparent that the JAK2 data is tilted towards larger colonies with more differentiated cells (Figure S3B).

To what extend can the model account for the behavior of the JAK2 mutant HSCs? Intriguingly, if we try to make use of the WT model to fit the experimental data for the JAK2 mutant cells, it is not possible to find a range of parameters, λ, κ and Γ, in which we can accurately reproduce the correct cell type averages at the 10 day timepoint. In particular, with

balanced probabilities for all proliferative cell types, $\Delta = 0$, the optimal fit of the model to the measured colony growth curve (Figure 5B) translates to a division rates $\lambda = 0.95$ per day and $\kappa = 2$ per day, a loss rate $\Gamma = 1.8$ per day, and a time offset increased from 1 day in WT to 1.5 days. With these parameters, a comparison of the cumulative clone size distribution with the experimental data shows a relative poor agreement. In particular, while the frequency of early undifferentiated cells (KSL and Lin-/non-KSL cells) is relatively good, lying within one standard deviation of the predicted model average distribution for 125 colonies, the cumulative distribution of viable cells is poorly recovered with the model showing a greater spread of colony sizes than the experimental data (Figure 5G). Therefore, to seek a better model for the JAK2 mutant, we will look for a minimal modification of the inferred dynamics of the WT system.

Inspired by the results of the serial transplantation assay, and the apparent tilt of the colony data towards differentiation, let us then suppose that cells at the apex of the hierarchy are now biased towards differentiation. For simplicity, let us begin by considering a 100% bias (i.e. $\Delta = 1/2$) for HSCs alone implying that, on division, the HSC immediately moves down the differentiation pathway. For cells lower in the hierarchy, we will assume that the fate behavior is equivalent to that of WT cells, with each generation perfectly balanced in its self-renewal potential, i.e. for these cells, the imbalance, $\Delta_n = \delta_n \equiv \Delta = 0$. Once again, adjusting the three parameters, we obtain the best fit of the model to the average total colony size over the timecourse (Figure 5A) and the average colony size by cell type at 10 days post-plating for $\lambda = 1.02 \pm 0.05$ per day, $\kappa = 1.83 \pm 0.1$ per day, and the same loss rate as WT, $\Gamma = 1.8$ per day. Crucially, for the same set of parameters, we obtain a much better agreement with the cumulative clone size distribution (Figure 5F,H), while the overlap of the joint colony size scatter is similar to that found for WT cells (Figure S4B). Therefore, in addition to the differentiation bias of the HSCs, these results suggest that the division rate of the JAK2 mutant cells is increased marginally over that of WT. Once again, if we allow a bias to develop in all of the differentiating cells, we find that the agreement of theory with the data is significantly compromised (data not shown).

Although this analysis indicates that the complete loss of self-renewal potential of the JAK2 mutant HSCs provides a better fit to the data than perfect balance, we must now address whether a partial bias of the HSCs towards differentiation may also be compatible with the experimental data. Indeed, if we fit the growth dynamics to a model with a 90% bias of HSCs towards differentiation (i.e. $\Delta = 0.4$), keeping the cells lower in the hierarchy balanced, the model provides an equally good fit to the cumulative size distributions with $\lambda = 1.00$ per

day, $\kappa = 1.86$ per day, and $\Gamma = 1.8$ per day (Figure S5A). By contrast, if we consider a 70% bias ($\Delta = 0.2$), with $\lambda = 0.98$ per day, $\kappa = 1.93$ per day, and $\Gamma = 1.8$ per day, the agreement is qualitatively less good (Figure S5B).

In summary, these simplified models suggest that JAK2 mutation compromises the survival potential of cells at the top of the stem cell hierarchy biasing them towards differentiation. Although this analysis is not sufficiently robust to rule out an additional bias towards differentiation in other generations near the top of the hierarchy, as with WT, the majority of cells lower in the hierarchy must retain a substantial degree of self-renewal potential to be consistent with the experimental data.

4 Ageing

Lastly, we turn to the question of ageing on the properties of the HSC compartment in the culture assay. In both the WT and JAK2 mutant, the data suggest that the population of all cell types is diminished slightly at the 10 day timepoint. At the same time, the 10 day timecourse shows that the entry of the HSCs into cycle is delayed slightly. This raises the question of whether the reduction in colony size represents a functional deficiency of these cells or is just a reflection of an increased activation period.

When compared to the colony data from young mice, the overlap of the cell type data from old mice (Figure S6A) shows that the pattern of cell growth and differentiation in WT cells is indeed similar. More significantly, if we suppose that HSCs from old mice incur a further 0.5 day delay in becoming activated – consistent with measurements of the 10 day timecourse (data not show) – the size distribution of the different cell types shows good agreement with the model prediction using the same division and loss rates (Figure S6B). An inspection of the data for JAK2 mutant HSCs leads to a very similar conclusion (data not shown).

Supplementary References

Clayton E., Doupé D.P., Klein A.M., Winton D.J., Simons B.D., and Jones P.H. (2007). A single type of progenitor cell maintains normal epidermis. Nature *446*, 185-189.

Haeno H., Levine R.L., Gilliland D.G., and Michor F. (2009). A progenitor cell origin of myeloid malignancies. Proc. Natl. Acad. Sci. USA *106*, 16616-16621.
